# Altered expression of somatostatin signaling molecules and clock genes in the hippocampus of subjects with substance use disorder

**DOI:** 10.3389/fnins.2022.903941

**Published:** 2022-09-07

**Authors:** Jake Valeri, Sinead M. O’Donovan, Wei Wang, David Sinclair, Ratna Bollavarapu, Barbara Gisabella, Donna Platt, Craig Stockmeier, Harry Pantazopoulos

**Affiliations:** ^1^Department of Psychiatry and Human Behavior, University of Mississippi Medical Center, Jackson, MS, United States; ^2^Program in Neuroscience, University of Mississippi Medical Center, Jackson, MS, United States; ^3^Department of Neuroscience, University of Toledo Medical Center, Toledo, OH, United States; ^4^Department of Medicine and Neurology, Brigham and Women’s Hospital, Boston, MA, United States

**Keywords:** substance use disorder, somatostatin, hippocampus, sleep, circadian

## Abstract

Substance use disorders are a debilitating group of psychiatric disorders with a high degree of comorbidity with major depressive disorder. Sleep and circadian rhythm disturbances are commonly reported in people with substance use disorder and major depression and associated with increased risk of relapse. Hippocampal somatostatin signaling is involved in encoding and consolidation of contextual memories which contribute to relapse in substance use disorder. Somatostatin and clock genes also have been implicated in depression, suggesting that these molecules may represent key converging pathways involved in contextual memory processing in substance use and major depression. We used hippocampal tissue from a cohort of subjects with substance use disorder (*n* = 20), subjects with major depression (*n* = 20), subjects with comorbid substance use disorder and major depression (*n* = 24) and psychiatrically normal control subjects (*n* = 20) to test the hypothesis that expression of genes involved in somatostatin signaling and clock genes is altered in subjects with substance use disorder. We identified decreased expression of somatostatin in subjects with substance use disorder and in subjects with major depression. We also observed increased somatostatin receptor 2 expression in subjects with substance use disorder with alcohol in the blood at death and decreased expression in subjects with major depression. Expression of the clock genes Arntl, Nr1d1, Per2 and Cry2 was increased in subjects with substance use disorder. Arntl and Nr1d1 expression in comparison was decreased in subjects with major depression. We observed decreased expression of Gsk3β in subjects with substance use disorder. Subjects with comorbid substance use disorder and major depression displayed minimal changes across all outcome measures. Furthermore, we observed a significant increase in history of sleep disturbances in subjects with substance use disorder. Our findings represent the first evidence for altered somatostatin and clock gene expression in the hippocampus of subjects with substance use disorder and subjects with major depression. Altered expression of these molecules may impact memory consolidation and contribute to relapse risk.

## Introduction

Substance use disorders (SUD) are a debilitating family of psychiatric disorders affecting approximately 4% of people in the United States each year, with 10% of adults suffering from SUD at some point during their lifetime (Grant et al., 2016). Relapse precipitated by exposure to environmental cues associated with drug taking is a major factor limiting recovery from SUD (Niaura et al., 1988; Carter and Tiffany, 1999; Weiss, 2005; Zironi et al., 2006; Janak and Chaudhri, 2010). The hippocampus is critically involved in encoding memories regarding environmental context including contextual cues related to fear and addiction (Kim and Fanselow, 1992; Phillips and LeDoux, 1992; Maren and Holt, 2000; Vorel et al., 2001; Rangel et al., 2014; Lehmann and Kenny, 2020; Felipe et al., 2021). Additionally, recent evidence suggests that hippocampal activity increases during reward anticipation, and that this increased activity is preceded by increased connectivity with the ventral tegmental area, indicating that dopamine modulation of the hippocampus by the VTA enhances hippocampal encoding during reward learning (Murty and Adcock, 2014). Furthermore, the hippocampus plays a major role in encoding predictability and uncertainty which are key factors in reward processing (Harrison et al., 2006; Hawley and Leasure, 2012; Huskinson, 2020).

Sleep disturbances are another major contributor to enhanced relapse risk (Brower et al., 1998, 2001; Brower and Hall, 2001; Mahfoud et al., 2009; Canellas and de Lecea, 2012). Sleep disturbances predict relapse (Brower, 2001), and disrupted sleep is a common complaint in people suffering from SUD (Mahfoud et al., 2009). Furthermore, recent preclinical evidence indicates that reduced slow wave sleep enhances cue induced reinstatement of alcohol seeking (Reeves-Darby et al., 2021), indicating that sleep disturbances may be a causal factor in relapse. In addition to the evidence for involvement of sleep disturbances in SUD, a growing number of studies highlight the involvement of the circadian system in this disorder (McClung et al., 2005; Falcon and McClung, 2009; Ozburn et al., 2013; DePoy et al., 2017; Edwards et al., 2018; Logan et al., 2018; Xue et al., 2021). Substances of abuse can disrupt circadian rhythms, and conversely, circadian rhythm disruption contributes to substance use (Parekh and McClung, 2015; DePoy et al., 2017; Logan et al., 2018).

The molecular circadian clock is found in nearly every cell throughout the brain and body. The molecular clock consists of a transcriptional-translational feedback loop that takes approximately 24 hours to complete (Reppert and Weaver, 2002; Ko and Takahashi, 2006). This process begins when Clock and Arntl form a heterodimer and through E-box enhancers, promote transcription of the clock components Per1,2,3 and Cry1,2 as well as many other “clock controlled” genes. Per and Cry accumulate in the cytoplasm, become phosphorylated, translocate back into the nucleus and repress their own transcription through their actions on Clock-Arntl (Reppert and Weaver, 2002; Ko and Takahashi, 2006). Additional molecules including GSK3β and NR1D1 interact with the molecular clock and modify the kinetics of this process (Reppert and Weaver, 2002; Ko and Takahashi, 2006). A key feature of the molecular clock is the ability of Clock-Arntl to promote transcription of many clock-controlled genes. Through this process, the molecular clock regulates the timing of a broad range of biological processes at the cellular level and in tissues, organs, systemic processes, and behavioral processes to optimally time these processes with the environment (Reppert and Weaver, 2002; Ko and Takahashi, 2006). Studies suggest that molecular clock rhythms in brain areas such as the hippocampus shift their rhythm to match the presence of biologically relevant stimuli such as availability of food or presence of predators (Verwey et al., 2007; Pantazopoulos et al., 2011).

Several studies have reported associations of clock gene polymorphisms with SUD (Yang et al., 2012; Edwards et al., 2018; Pabalan et al., 2021). Although circadian rhythms are controlled by clock genes in the suprachiasmatic nucleus (SCN), clock genes are present throughout the brain and body and coordinate various biological and neural functions including sleep, stress and memory (Hastings, 1998; Reppert and Weaver, 2002; Takahashi, 2017; Bolsius et al., 2021). Rhythms in clock molecule expression have been reported in several brain regions outside of the SCN in rodents and humans, including the hippocampus (Pantazopoulos et al., 2011, 2017; Harbour et al., 2013; Li et al., 2013; Chen et al., 2016; Ketchesin et al., 2021; Logan et al., 2022). Clock molecule rhythms in these regions are entrained by biologically relevant environmental signals such as food availability or fearful stimuli (Amir and Stewart, 2009; Pantazopoulos et al., 2011; Page et al., 2020; Nishide et al., 2021), suggesting that molecular clock rhythms regulate the timing of processes in a brain region specific manner to optimally respond to biologically relevant environmental factors.

How sleep and circadian rhythm disturbances impact specific neurotransmitter systems that in turn contribute to SUD and relapse is a key question in developing treatment strategies. Hippocampal somatostatin (SST) signaling represents a promising target for therapeutic development. Increasing evidence suggests that SST signaling is involved in several key processes in SUD (for review see Robinson and Thiele (2020)). Preclinical studies suggest that SST signaling is involved in regulating several reward-related behaviors including food intake and cocaine and alcohol consumption (Fuhrmann et al., 1986; Mancillas et al., 1986; Barrios et al., 1990; Andrade et al., 1992; Yuferov et al., 2003; Stengel et al., 2010b). SST administration increases dopamine release in the striatum (Hathway et al., 1998) and SST and SST receptor expression is altered in several brain regions including the hippocampus following exposure to alcohol or cocaine (Fuhrmann et al., 1986; Mancillas et al., 1986; Barrios et al., 1990; Andrade et al., 1992; Yuferov et al., 2003). Furthermore, recent findings demonstrate a key role for SST signaling in the hippocampus in regulating memory consolidation during sleep (Delorme et al., 2021). Thus, SST signaling is at the intersection of sleep, circadian rhythm, and reward processes involved in SUD.

Despite these findings, there is currently a lack of information regarding rhythms of clock genes and SST/SSTR expression in the hippocampus of individuals with SUD. Subjects with SUD display a high degree of comorbidity with major depressive disorder (MDD), making it challenging to identify neurobiological factors uniquely underlying SUD (Davis et al., 2008; Grant et al., 2016; Substance Abuse and Mental Health Services Administration [SAMHSA], 2020). Furthermore, potential effects of confounding factors including presence or absence of drugs of abuse and psychiatric medications in the blood at time of death have made it challenging to examine molecular pathology in postmortem studies of SUD and MDD. In the present study, we used a cohort of subjects with comorbid SUD and MDD and subjects with MDD or SUD only, as well as psychiatrically-normal control subjects, in combination with extensive information regarding confounding factors and toxicology reports to address these challenges. People with SUD often use multiple substances, possibly reflecting in part shared underlying genetic factors (Hatoum et al., 2021). Our cohort of subjects with SUD and comorbid SUD and MDD consist of subjects with polysubstance use, reflecting the more typical “real-world” pattern of drug use by people with SUD. As a first step in examining the sleep related molecular pathways affected in the hippocampus of subjects with SUD associated with sleep disturbances, we first tested the hypothesis that subjects with SUD in our cohort have a higher prevalence of sleep disturbances. Next, we tested the hypothesis that SST and SSTR2 expression is altered in the hippocampus of subjects with SUD. Lastly, we tested the hypothesis that expression of clock genes and diurnal expression rhythms are enhanced in the hippocampus of subjects with SUD.

## Materials and methods

### Human subjects

Institutional Review Boards of the University of Mississippi Medical Center, Jackson, MS and the University Hospitals Cleveland Medical Center, Cleveland, OH, had approved all the procedures and were in accordance with the Declaration of Helsinki. Informed consent from the legally-defined next-of-kin was obtained for the collection of tissue, medical records, and retrospective psychiatric interviews. Structured Clinical Interview for DSM-IV Axis I Disorders (First et al., 1995) was administered by a Master-level social worker to knowledgeable informants of the subjects, as outlined in Zhu et al. (2012). To determine subjects’ psychopathology, a board-certified clinical psychologist and a board-certified psychiatrist independently reviewed the diagnostic interview scoring notation, the medical examiner’s report, any prior medical records, and a comprehensive narrative that summarized all scores of information about each subject. The social worker, the clinical psychologist, and the psychiatrist reached a consensus on the diagnosis. Cause of death was determined by the medical examiner. Subjects in either group who met Diagnostic and statistical manual of mental disorders (DSM-IV; American Psychiatric Association [APA], 2000) criteria for substance use disorder (SUD) and/or major depressive disorder (MDD) diagnosis were included for this study. The presence of psychotropic medications and substances of abuse in blood and urine at death was determined by the medical examiner’s office. Toxicology reports as reported by the medical examiner were used to determine presence or absence of ethanol, cocaine, opioids, SSRIs, antidepressants, antipsychotics, lithium, and benzodiazepines in the blood at death. Exposure to psychiatric medications in the last month of life was estimated as described in Sullivan et al. (2018). Detailed subject demographic information is provided in Supplementary Tables 1–16. Information regarding potential confounding variables including time of death calculated as zeitgeber time (ZT), postmortem interval, tissue pH, race, age and sex, history of substance use including nicotine, alcohol, cocaine, opioids, and marijuana, pharmacological treatment, sleep quality, suicide, duration of alcohol use disorder, duration of MDD, and mood symptom severity in the last two weeks of life were obtained from medical records, police reports, medical examiner’s reports, and family interviews. Duration of alcohol use was estimated from informant interviews and medical records by estimating the onset of alcohol use disorder and established recovery if any, along with the age of the subject at death if no recovery was established. Alcohol or drug (AUD or SUD) characterizations included abuse and dependence according to DSM-IV criteria. Determination of AUD or SUD was conducted by grouping both abuse and dependence according to DSM-IV criteria. Subjective quality of sleep was obtained from medical records and family questionnaires for each subject when available and characterized as normal sleep (no history of sleep disturbances), decreased sleep (history of trouble sleeping) or increased sleep (history of excessive sleep). Mood symptom severity in the last two weeks of life was determined and noted for each subject by the UMMC Postmortem Brain Core. Mood symptom severity as well as comorbidity with obsessive-compulsive disorder, anxiety disorder, or personality disorder were evaluated by a board-certified clinical psychologist and a board-certified psychiatrist who independently reviewed the diagnostic interview scoring notation, the medical examiner’s report, any prior medical records, and a comprehensive narrative that summarized all scores of information about each subject. Comorbidity with obsessive-compulsive disorder, anxiety disorders, or personality disorders was classified as presence or absence of OCD, anxiety disorder, or personality disorder, respectively for ANCOVA analysis. Lifetime history of substance use was determined from medical records and family interviews and characterized as yes or no based on history of chronic use for each substance including alcohol, cocaine, opioids, and marijuana. Furthermore, subjective ratings of lifetime alcohol and nicotine intake were determined from medical records and family interviews and rated from 0 (none) to 4 (high). These ratings were performed by one investigator (HP) prior to data collection while blind to the results of this study. Presence or absence of recent history of calcium channel blockers was determined from medical records and family interviews.

### Tissue preparation

Hippocampi were derived from postmortem brains collected at autopsy at the Cuyahoga County Medical Examiner’s Office, Cleveland, OH. Tissues were dissected and rapidly frozen in 2-methylbutane on dry ice without fixation and kept in dry ice before permanent storage at –80°C. Postmortem hippocampus samples from 20 subjects diagnosed with SUD, 24 with comorbid SUD and MDD, 20 with MDD, and 20 psychiatrically-normal control donors were obtained from the University of Mississippi Medical Center Postmortem Brain Core. Hippocampi were cut into 14 μm thick sections using a cryostat (Leica CM3050S) and mounted on Fisher Superfrost slides.

### Quantitative polymerase chain reaction

qPCR was conducted as previously described (O’Donovan et al., 2015, 2018; Abel et al., 2021). Briefly, RNA was extracted from 14 μm hippocampal sections using the RNeasy Minikit (Qiagen, NL) according to the manufacturer’s instructions, the concentration was assayed (NanodropOne) and equalized to 7.4 ng/ul. Complementary DNA (cDNA) was synthesized using a High-Capacity cDNA Reverse Transcription Kit (Applied Biosystems, Foster City, CA, United States). For each reaction, 0.5 μl of cDNA (1:3 diluted) was placed in a 10 μL reaction containing 5 μL of SYBR Green PCR Master Mix (Applied Biosystems) and 10 pmol of each primer (Invitrogen, United States) or FastStart TaqMan Probe Master and 1x Taqman primers (ThermoFisher). The primers used are listed in Table 1. Custom-made assays were tested for specificity and resulted in a single band of expected size, which was confirmed with excision and sequencing. All reactions were performed in triplicate using 384-well optical reaction plates (Life Technologies, United States) on an Applied Biosystems detection system (QuantStudio 5, Applied Biosystems, Life Technologies, United States). Reactions were performed with an initial ramp time of 10 min at 95°C, and 40 subsequent cycles of 15 s at 95°C and 1 min at 60°C. For negative controls for the qPCR reactions, non-template control (cDNA was omitted) and no-RT control (reverse transcriptase excluded from cDNA synthesis reaction) were run on each plate. Relative concentrations of the transcripts of interest were calculated with comparison to a standard curve made with dilutions of cDNA from a pooled sampling of all the subjects according to established methods (Cikos et al., 2007). The standard curve is composed of a calibrator sample – a pool sample of subjects from all 4 groups used in the study. A standard curve is generated for each gene of interest and reference gene. The gene expression in all samples is calculated in relation to the standard curve, and then the genes of interest are normalized to the geomean of the reference (housekeeping) genes. Values for the transcripts of interest were normalized to the geometric mean of B2M, ACTB, GAPDH and PPIA values for the same samples according to established methods (Vandesompele et al., 2002). Data were collected by QuantStudio Design and Analysis software v1.5.1.

**TABLE 1:** Table of primer probes.

Probes	Forward	Reverse
Experimental		
*Arntl (Bmal1)*	CATGCTGCCCTCTGGAGAA	GCGATGACCCTCTTATCCTGT
*Cry2*	ACCGGGGACTCTGTCTACTG	GCCTGCACTGCTCATGCT
*Gsk3*β	TAACCCAGGGAGGTCAATAA	TAGAGCATGTGTGCCTGAGT
*Rev-erb*α	CGGCGATCGCAACCTCTAGT	AGGTGATGACGCCACCTGT
*Per2*	TTCTCCCATTCGGTTTCG	CCTGACTTTGTGCCTCCC
*Sst*	TGAACCCAACCAGACGGAGA	ATAGCCGGGTTTGAGTTAGCA
*Sstr2*	GCTGGCTGGAACTAGCCTAA	ACACCACAGAGCCATTGAGG
Loading controls		
*Actb*	GTCATTCCAAATATGAGATGCGT	GCTATCACCTCCCCTGTGTG
*Ppia*	ATGGTCAACCCCACCGTGTTCTTCG	CGTGTGAAGTCACCACCCTGACACA
*Gapdh*	TCGACAGTCAGCCGCATCT	AGTTAAAAGCAGCCCTGGTGA
*B2m*	GTGGGATCGAGACATGTAAGC	AGCAAGCAAGCAGAATTTGGAAT

### Statistical analysis

The goal of our study was to test for diagnosis group differences in gene expression measures, taking into account potential effects of covariates. Differences between groups relative to the main outcome measures were assessed for statistical significance using stepwise linear regression analysis of covariance (ANCOVA) as our primary analysis. Logarithmic transformation was uniformly applied to all values because data were not normally distributed. Statistical analyses were performed using JMP Pro v16.1.0 (SAS Institute Inc., Cary, NC). Potential confounding variables (Tables 1, 2) were tested systematically for their effects on main outcome measures and included in the model if they significantly improved goodness-of-fit. Covariates found to significantly affect outcome measures are reported. Subjects with SUD, subjects with MDD/SUD, and subjects with MDD were first compared separately with psychiatrically-normal controls. Subsequently, the 4 groups were considered together to test for differences between diagnostic groups and Bonferroni corrected for multiple comparisons.

**TABLE 2:** Summary table of diagnosis group comparisons.

	Diagnosis	F ratio	*p* value	Adj. least sq. mean	Standard error	Covariates
*Sst*	SUD	**6.53**	**0.007**	Con: 0.10; SUD: –0.26	Con: 0.10; SUD: 0.07	Ethanol in tox, tissue pH
	MDD	**4.23**	**0.035**	Con: –0.06; MDD: –0.21	Con: 0.03; MDD: 0.05	Age, duration MDD, PMI
	MDD/SUD	0.95	0.46	Con: –0.21; MDD/SUD: 0.13	Con: 0.08; MDD/SUD: 0.05	Age, suicide
*Sst2r*	SUD	4.03	0.99	Con: 0.15; SUD: 0.15	Con: 0.05; SUD: 0.04	Ethanol in tox
	MDD	**3.06**	**0.02**	Con: 0.10; MDD: –0.04	Con: 0.03; MDD: 0.04	Smoking, ZT time
	MDD/SUD	1.15	0.29	Con: 0.08; MDD/SUD: 0.13	Con: 0.04; MDD/SUD: 0.03	*None*
*Arntl*	SUD	**5.03**	**0.03**	Con: –0.08; SUD: 0.06	Con: 0.04; SUD: 0.05	ZT time, ZT time*DDx
	MDD	**5.22**	**0.01**	Con: –0.03; MDD: –0.34	Con: –0.05; MDD: –0.09	ZT time, duration of MDD, age
	MDD/SUD	3.50	0.69	Con: 0.09; MDD/SUD: 0.12	Con: 0.09; MDD/SUD: 0.07	ZT time, ZT time*DDx, opioids in tox
*Per2*	SUD	**5.18**	**0.001**	Con: 0.21; SUD: 0.56	Con: 0.08; SUD: 0.10	ZT time, calcium channel blockers, alcohol
	MDD	2.81	0.17	Con: 0.20; MDD: 0.30	Con: 0.07; MDD: 0.07	Nicotine, calcium channel blockers, ZT time
	MDD/SUD	**3.13**	**0.007**	Con: 0.18; MDD/SUD: 0.49	Con: 0.09; MDD/SUD: 0.11	ZT time, sleep dist., calcium channel blockers, antipsychotics in last month of life
*Cry2*	SUD	**5.45**	**0.02**	Con: 0.01; SUD: 0.36	Con: 0.06; SUD: 0.09	ZT time, ZT time*DDx, duration of AUD
	MDD	4.83	0.57	Con: 0.32; MDD: 0.30	Con: 0.05; MDD: 0.05	ZT time, calcium channel blockers, alcohol
	MDD/SUD	5.13	0.87	Con: 0.53; MDD/SUD: 0.53	Con: 0.10; MDD/SUD: 0.08	ZT time, opioids and cocaine in tox
*Nr1d1*	SUD	**3.81**	**0.02**	Con: –0.35; SUD: –0.17	Con: 0.06; SUD: 0.04	Cocaine in tox
	MDD	0.36	0.74	Con: –0.27; MDD: –0.25	Con: 0.04; MDD: 0.05	ZT time
	MDD/SUD	**8.68**	**0.03**	Con: 0.19; MDD/SUD: –0.03	Con: 0.09; MDD/SUD: 0.08	ZT time, duration of MDD, sex, smoking, opioids and cocaine in tox
*Gsk3*β	SUD	**7.21**	**0.002**	Con: 0.27; SUD: 0.05	Con: 0.06; SUD: 0.04	ZT time, ZT time*DDx, PMI, ethanol and opioids in tox
	MDD	0.39	0.40	Con: 0.07; MDD: 0.02	Con: 0.04; MDD/SUD: 0.04	ZT time
	MDD/SUD	**5.28**	**0.0003**	Con: 0.43; MDD/SUD: –0.33	Con: 0.09; MDD/SUD: 0.09	ZT time, duration of AUD, suicide

PMI, Postmortem interval; ZT, zeitgeber time. Bold values represent statistically significant differences in comparison to control subjects.

We conducted a secondary analysis of diurnal rhythms of gene expression in order to obtain additional information regarding the effects of time of death on gene expression measures, as time of death was a significant covariate in many of our ANCOVA models. To analyze the relationship of gene expression with time of death (TOD), we employed established methods to normalize TOD measures to zeitgeber time (ZT) scale according to sunrise time at local time and season at place of death, accounting for potential variations in local photoperiod for each subject (Chen et al., 2016; Seney et al., 2019). ZT0 represents sunrise time at at local time and season at place of death. ZT values greater than zero represent number of hours after sunrise, and negative ZT values represent hours before sunrise according to previous studies (Chen et al., 2016; Seney et al., 2019; Ketchesin et al., 2021; Logan et al., 2022). We analyzed plots of gene expression measures by TOD according to methods previously shown to detect similar relationships in postmortem studies (Zhou et al., 2001; Hofman, 2003; Li et al., 2013; Seney et al., 2019; Ketchesin et al., 2021). Non-linear regression models were used to detect diurnal gene expression as a function of adjusted TOD using SAS V9.4 PROC NLINMIXED. A sinusoidal curve was fit as described in previous work (Li et al., 2013; Chen et al., 2016; Seney et al., 2019) to compare between diagnosis groups (SUD and MDD/SUD) and control subjects on measures of rhythmicity: *R*^2^ values, mesor values, phase (estimated peak time of expression), and amplitude (magnitude of peak expression). *R*^2^ and mesor values are reported for each outcome measure in each diagnosis group, and phase and amplitude are reported for each measure with significant *R*^2^ value.

## Results

Our primary analysis focused on testing differences between control subjects and each diagnosis group (SUD, MDD/SUD, and MDD) using stepwise linear regression ANCOVA. We note that for diagrams depicting comparison of overall expression levels per group, scatterplots depicting individual values, means and confidence intervals do not depict values adjusted for effects of covariates. However, the *p*-values for each graph are adjusted for significant effects of covariates derived from ANCOVA models.

### Sleep disturbances in substance use disorders and major depressive disorder subjects

**FIGURE 1:**
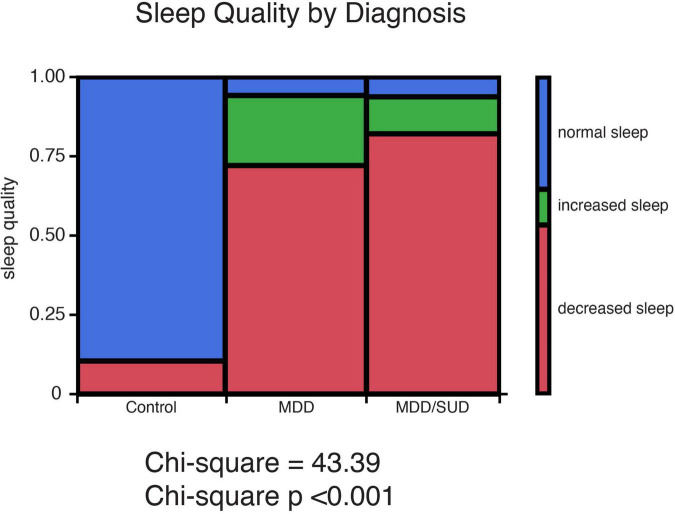
History of sleep disturbances in subjects with SUD and subjects with MDD. Sleep quality ratings representing presence or absence of history of sleep disturbances were obtained from medical records and family interviews of each donor. Sleep quality was characterized as normal sleep, decreased sleep, or increased sleep. Chi-square analysis revealed a significant effect of diagnosis group (Controls, MDD or SUD and SUD with MDD) on sleep quality. Subjects with MDD and subjects with MDD/SUD had higher prevalence of decreased sleep as well as a smaller group of subjects with increased sleep.

Chi-square analysis of subjective sleep quality revealed a significant effect of diagnosis group (Controls, MDD or SUD and SUD with MDD) on sleep quality (Figure 1). Subjects with MDD and subjects with MDD/SUD had higher prevalence of decreased sleep as well as a smaller group of subjects with increased sleep. Sleep quality data were not available for a number of subjects in the SUD only group, thus these subjects were considered together with the comorbid MDD/SUD subjects for chi square analysis of sleep disturbances. Despite this limitation, all subjects in the SUD group who had available sleep quality information had a history of sleep disturbances, in line with the findings in the comorbid MDD/SUD group and findings reported in the general population relapse (Brower et al., 1998, 2001; Brower and Hall, 2001; Mahfoud et al., 2009; Canellas and de Lecea, 2012).

### Altered somatostatin signaling in substance use disorders and major depressive disorder subjects

#### Somatostatin

**FIGURE 2:**
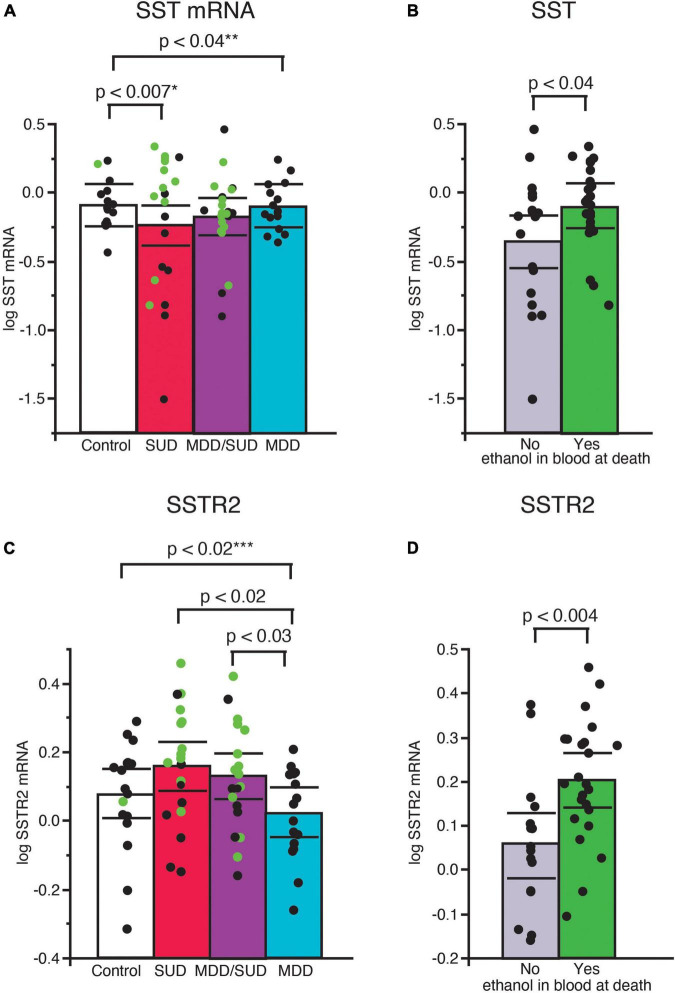
Altered SST signaling in the hippocampus of subjects with SUD and subjects with MDD. Scatterplots depicting normalized mRNA expression for Sst in each diagnosis group **(A)**. Green circles represent subjects with alcohol in the blood at death. SST mRNA expression was significantly greater in subjects with SUD compared to control subjects, (*p* < 0.0007, *adjusted for effects of alcohol in the blood at death and pH). Subjects with alcohol in the blood at death had significantly greater Sst mRNA expression than subjects without alcohol in the blood **(B)**. Subjects with MDD/SUD had no significant difference in Sst mRNA expression when compared to control subjects (adjusted for significant effects of age and suicide). A significant decrease in Sst mRNA expression was detected in subjects with MDD (*p* < 0.04, **adjusted for effects of age, duration of illness, and PMI). No significant differences in Sstr2 mRNA expression were detected in subjects with SUD compared to control subjects when adjusted for effect of alcohol in the blood at death **(C)**. Subjects with alcohol in the blood at death had significantly greater expression of SSTR2 mRNA **(D)**. Subjects with MDD had significantly lower Sstr2 mRNA expression compared to control subjects (*p* < 0.02, ***adjusted for effects of smoking history and ZT time). Subjects with MDD also displayed lower Sstr2 mRNA expression in comparison to subjects with SUD or subjects with MDD/SUD **(C)**. Significance values are derived from stepwise linear regression models. Scatterplots show the mean (histogram) and 95% confidence intervals (black lines).

Sst mRNA expression was significantly decreased in subjects with SUD (*p* < 0.007; adjusted for significant effects of ethanol in the blood at death and tissue pH; Figure 2A). Subjects with alcohol in the blood at death had significantly greater Sst mRNA expression compared to subjects without alcohol (*p* < 0.04; Figure 2B). Sst mRNA expression was also significantly decreased in subjects with MDD only compared to control subjects (*p* < 0.04; Figure 2A; adjusted for significant effects of age, duration of illness, and PMI). In comparison, Sst mRNA expression in subjects in the MDD/SUD group was not significantly different when compared to control subjects (Figure 2A; adjusted for significant effects of age and suicide).

#### Sstr2

Sstr2 mRNA expression was not altered in subjects with SUD compared to control subjects when adjusted for a significant effect of alcohol in the blood at death (Figure 2C). Sstr2 mRNA expression was significantly greater in subjects with alcohol in the blood at death (Figure 2D). In comparison, Sstr2 mRNA expression was significantly decreased in subjects with MDD only compared to control subjects (*p* < 0.02) adjusted for significant effects of smoking history and TOD (Figure 2C). No differences in Sstr2 mRNA expression were detected between subjects with comorbid MDD/SUD and control subjects (Figure 2C). No significant correlations with Sst or Sstr2 mRNA expression was detected with duration of alcohol use (Supplementary Figure 1).

### Altered clock gene expression in substance use disorders and major depressive disorder subjects

#### ARNTL

**FIGURE 3:**
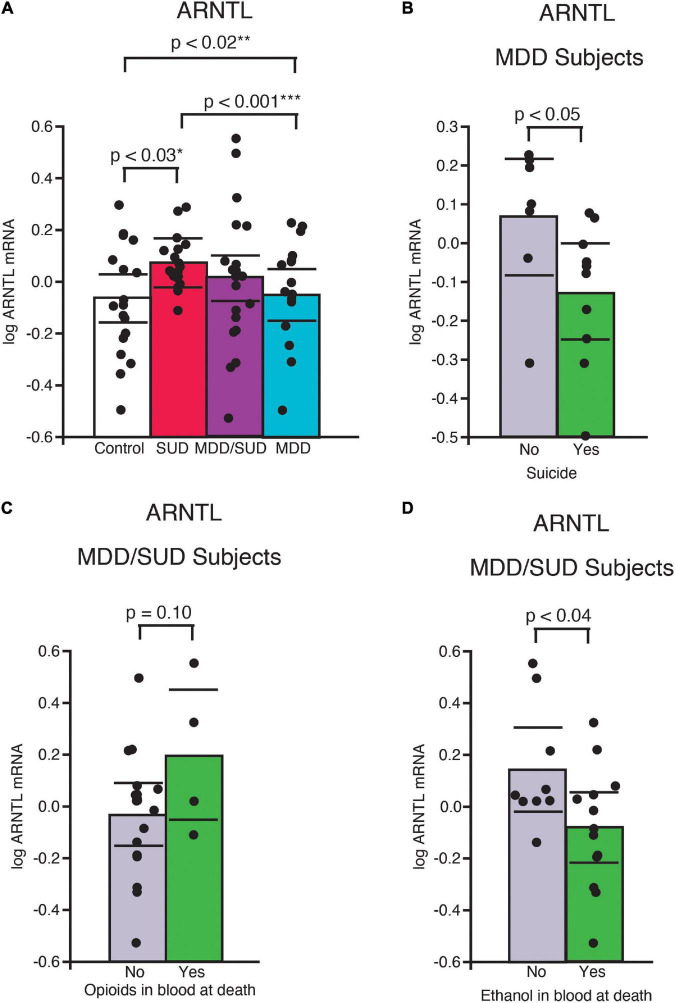
ARNTL expression is differentially altered in the hippocampus of subjects with SUD and subjects with MDD. **(A)** Arntl mRNA expression was significantly greater in subjects with SUD compared to control subjects (*p* < 0.03, *adjusted for significant effects of ZT time and interaction of ZT time with diagnosis). A significant decrease in Arntl mRNA expression was observed in subjects with MDD only compared to control subjects (*p* < 0.02, **adjusted for significant effects of ZT time, duration of illness, and age). Subjects with MDD also displayed lower Arntl expression compared to subjects with SUD (*p* < 0.001, ***adjusted for significant effects of ZT time and duration of MDD). Subjects with MDD who died by suicide had significantly less ARrntl mRNA expression than subjects with MDD who died from other causes **(B)**. Subjects with MDD/SUD with opioids in the blood at death had a non-significant trend toward increased Arntl expression **(C)** compared to significantly less Arntl expression in subjects with ethanol in the blood at death **(D)**. Significance values are derived from stepwise linear regression models. Scatterplots show the mean (histogram) and 95% confidence intervals (black lines).

Arntl mRNA expression was significantly greater in subjects with SUD only compared to control subjects (*p* < 0.03, Figure 3A; adjusted for significant effects of ZT time and interaction of ZT time with diagnosis). Subjects with SUD only also displayed significantly greater Arntl expression compared to subjects with MDD only (*p* < 0.001; Figure 3A; adjusted for significant effects of ZT time and duration of MDD). In comparison, a significant decrease in Arntl mRNA expression was observed in subjects with MDD only compared to control subjects (*p* < 0.02, Figure 3A; adjusted for effects of ZT time, duration of illness, and age). Lower Arntl mRNA was also observed in subjects with MDD who died by suicide compared to subjects with MDD who died from other causes (Figure 3B). No significant difference in Arntl expression was detected between subjects with comorbid MDD/SUD and control subjects when ZT time, interaction of ZT time and diagnosis, and opioids in the blood at death were included in the model (Figure 3A). In the MDD/SUD group, subjects with opioids in the blood at death had a trend toward an increase in Arntl expression (Figure 3C), whereas subjects with ethanol in the blood at death had significantly lower Arntl expression (Figure 3D).

#### NR1D1

**FIGURE 4:**
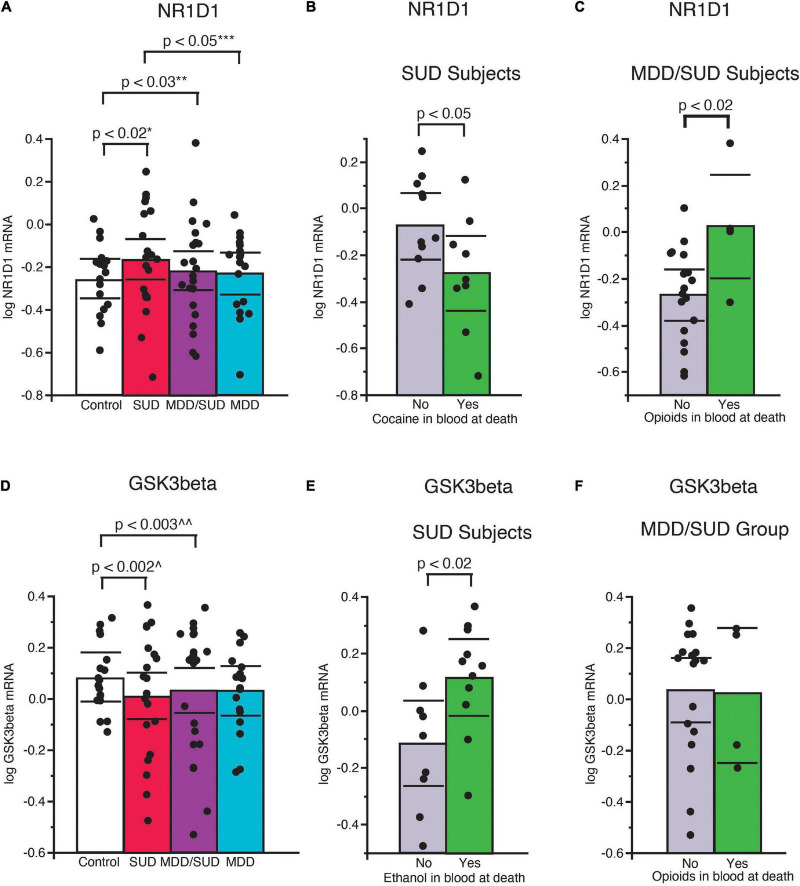
Altered expression of NR1D1 and GSK3β in the hippocampus of subjects with SUD. **(A)** Significantly greater Nr1d1 mRNA expression was observed in subjects with SUD compared to control subjects (*p* < 0.02, *adjusted for significant effect of cocaine in the blood at death). Subjects with SUD with cocaine in the blood at death had significantly less Nr1d1 mRNA expression **(B)**. Subjects with MDD/SUD had significantly lower Nr1d1 expression compared to control subjects (*p* < 0.03; **adjusted for significant effects of ZT time, duration of MDD, sex, opioids in the blood at death, smoking, and cocaine in the blood at death). Subjects with MDD/SUD with opioids in the blood at death had significantly higher Nr1d1 expression than subjects **(C)**. Subjects with MDD only had no significant difference in Nr1d1 expression when compared to control subjects **(A)**. Subjects with MDD only had significantly lower expression compared to subjects with SUD (*p* < 0.05, ***adjusted for significant effect of cocaine in the blood at death). Scatterplots depicting normalized mRNA expression for Gsk3β **(D)**. Gsk3β mRNA expression was significantly decreased in subjects with SUD (*p* < 0.002; ^adjusted for significant effects of ZT time, interaction of ZT time and diagnosis, PMI, ethanol in the blood at death **(E)**, and opioids in the blood at death) **(F)**. Significance values are derived from stepwise linear regression models. Scatterplots show the mean (histogram) and 95% confidence intervals (black lines). ^^Adjusted for effects of duration of alcohol use disorder and suicide.

A significant increase in Nr1d1 mRNA expression was observed in subjects with SUD compared to control subjects (*p* < 0.02, Figure 4A; adjusted for significant effect of cocaine in the blood at death). Subjects in the SUD group with cocaine in the blood at death had significantly lower Nr1d1 expression (Figure 4B). No significant difference in Nr1d1 expression was observed in subjects with MDD only when compared to control subjects. Subjects with MDD only had significantly lower expression compared to subjects with SUD (Figure 4A; *p* < 0.05, adjusted for significant effect of cocaine in the blood at death). Subjects with comorbid MDD/SUD had significantly lower Nr1d1 expression compared to control subjects (*p* < 0.03; Figure 4A; adjusted for significant effects of ZT time, duration of MDD, sex, opioids in the blood at death, smoking, and cocaine in the blood at death). Subjects in this group with opioids in the blood at death had significantly higher expression of Nr1d1 (Figure 4C).

#### GSK3β

Decreased expression of Gsk3β was detected in subjects with SUD compared to control subjects (*p* < 0.002; Figure 4D; adjusted for significant effects of ZT time, interaction of ZT time and diagnosis, PMI, ethanol in the blood at death, and opioids in the blood at death). Subjects in this diagnosis group with ethanol in the blood at death had significantly greater Gsk3β expression (Figure 4E). No difference in Gsk3β expression was observed between subjects with MDD only and control subjects (Figure 4D; adjusted for effect of ZT time). Subjects with comorbid MDD/SUD had significantly lower levels of Gsk3β expression compared to control subjects (*p* < 0.003; Figure 4D; adjusted for significant effects of ZT time, duration of alcohol use disorder, and suicide).

#### Per2 and Cry2

**FIGURE 5:**
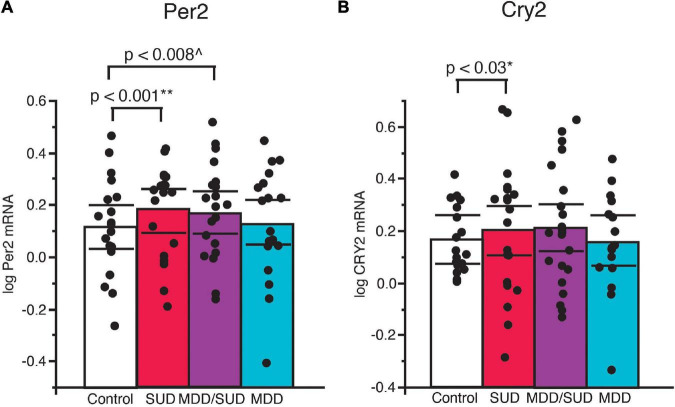
Altered Per2 and Cry2 expression in the hippocampus of subjects with SUD. **(A)** ANCOVA analysis detected increased Per2 mRNA expression in subjects with SUD (*p* < 0.001; **adjusted for effects of ZT time, calcium channel blockers, and rating of lifetime alcohol exposure). Subjects with comorbid MDD/SUD also displayed significantly increased Per2 expression (*p* < 0.008; ^adjusted for effects of ZT time, history of sleep disturbances, calcium channel blockers, and amount of antipsychotics in the last month of life) **(A)**. No differences in Per2 expression were detected in subjects with MDD. **(B)** Increased Cry2 expression was also detected in subjects with SUD (*p* < 0.03; *adjusted for effects of ZT time, interaction of ZT time with diagnosis, and duration of alcohol use disorder). No differences in Per2 expression were detected in subjects with MDD/SUD or subjects with MDD. Significance values are derived from stepwise linear regression models. Scatterplots show the mean (histogram) and 95% confidence intervals (black lines).

Increased Per2 mRNA expression was detected in subjects with SUD (*p* < 0.001; Figure 5A; adjusted for significant effects of ZT time, calcium channel blockers, and rating of lifetime alcohol exposure). Significantly greater Per2 expression also was observed in subjects with comorbid MDD/SUD compared to control subjects (*p* < 0.008; Figure 5A; adjusted for significant effects of ZT time, history of sleep disturbances, calcium channel blockers, and exposure to antipsychotics in the last month of life). No differences in Per2 expression were detected in subjects with MDD compared to any of the other diagnosis groups (Figure 5A). A similar significant increase in gene expression was observed for Cry2 in subjects with SUD (*p* < 0.03; Figure 5B; adjusted for significant effects of ZT time, interaction of ZT time with diagnosis, and duration of alcohol use disorder. No changes in Cry2 expression were observed in subjects with comorbid MDD/SUD or MDD when compared to other diagnosis groups (Figure 5B).

Table 2 summarizes the results of our diagnosis group comparisons along with adjusted means and covariates used in each model.

#### Diurnal molecular expression rhythms

We conducted a secondary analysis to test diurnal rhythms of gene expression on the same measures from our primary analysis because ZT time was a significant covariate in many of our ANCOVA models. The goal of this secondary analysis is to provide additional information regarding the effects of ZT time on the diagnosis group comparisons detected in ANCOVA models, which incorporate many covariates in addition to ZT time. We used non-linear regression models were used to detect diurnal gene expression as a function of subject TOD normalized to ZT scale in order to provide more information into the potential effects of ZT time in diagnosis group differences observed with ANCOVA models (Chen et al., 2016; Seney et al., 2019). ZT0 represents sunrise time at at local time and season at place of death. ZT values greater than zero represent number of hours after sunrise, and negative ZT values represent hours before sunrise. *R*^2^ values with *p*-values less than 0.05 represent transcripts with significantly rhythmic expression. Amplitude and phase estimates were also determined for each outcome measure in each group that had significant *R*^2^ values. Statistical comparisons between groups were conducted on measures in which individual group *R*^2^ values achieved a *p*-value of less than 0.05. Table 3 includes *R*^2^ values and mesor values for each outcome measure in each group as well as amplitude and phase estimates for outcome measures in groups with significant *R*^2^ values. Sampling intervals and distribution of ZT values were limited for diurnal analysis of expression rhythms, and ZT values were not equally distributed between diagnosis groups. ZT values for subjects with MDD only were available for only 12 out of 20 subjects and were concentrated in the first half of the 24-hour ZT scale, thus diurnal rhythm analysis was not conducted for this group. Despite these limitations, our gene expression analysis consisting of accounting for potential effects of ZT in ANCOVA models allows us to correct for potential effects of ZT when comparing gene expression levels between diagnosis groups. Furthermore, our ZT diurnal expression rhythm analysis provides insight into potential alterations in hippocampal molecular diurnal rhythms in SUD and MDD.

**TABLE 3:** Table of diurnal rhythm analysis results.

Outcome	Group	*R* ^2^	One-sided *P*-value	Mesor	Phase estimate	Phase standard error	Amplitude estimate	Amplitude standard error
log *Sstr2* mRNA	Control	0.36	0.030	0.08	14.13	1.50	0.11	0.05
	MDD/SUD	0.02	*n.s.*	0.11	*n.s.*	*N/A*	*n.s.*	*N/A*
	SUD	0.54	0.023	0.07	–4.31	1.21	0.19	0.07
log *Cry2* mRNA	Control	0.08	n.s.	0.16	*n.s.*	*N/A*	*n.s.*	*N/A*
	MDD/SUD	0.14	*n.s.*	0.30	*n.s.*	*N/A*	*n.s.*	*N/A*
	SUD	0.26	*n.s.*	0.11	*n.s.*	*N/A*	*n.s.*	*N/A*
log *Gsk3*β mRNA	Control	0.45	0.009	0.059	16.10	1.61	0.14	0.07
	MDD/SUD	0.13	*n.s.*	0.06	*n.s.*	*N/A*	*n.s.*	*N/A*
	SUD	0.38	0.05	–0.067	–1.89	0.85	0.29	0.09
log *Nr1d1* mRNA	Control	0.08	*n.s.*	–0.27	*n.s.*	*N/A*	*n.s.*	*N/A*
	MDD/SUD	0.35	0.029	–0.27	4.12	1.39	0.20	0.06
	SUD	0.47	0.029	–0.28	2.16	1.43	0.22	0.08
log *Per2* mRNA	Control	0.00	*n.s.*	0.10	*n.s.*	*N/A*	*n.s.*	*N/A*
	MDD/SUD	0.04	*n.s.*	0.14	*n.s.*	*N/A*	*n.s.*	*N/A*
	SUD	0.04	*n.s.*	0.22	*n.s.*	*N/A*	*n.s.*	*N/A*
log *Sst* mRNA	Control	0.24	*n.s.*	–0.08	*n.s.*	*N/A*	*n.s.*	*N/A*
	MDD/SUD	0.14	*n.s.*	–0.12	*n.s.*	*N/A*	*n.s.*	*N/A*
	SUD	0.24	*n.s.*	–0.45	*n.s.*	*N/A*	*n.s.*	*N/A*
log *Arntl* mRNA	Control	0.44	0.013	–0.08	13.41	1.05	0.21	0.06
	MDD/SUD	0.27	*n.s.*	0.01	*n.s.*	*N/A*	*n.s.*	*N/A*
	SUD	0.20	*n.s.*	0.05	*n.s.*	*N/A*	*n.s.*	*N/A*

**FIGURE 6:**
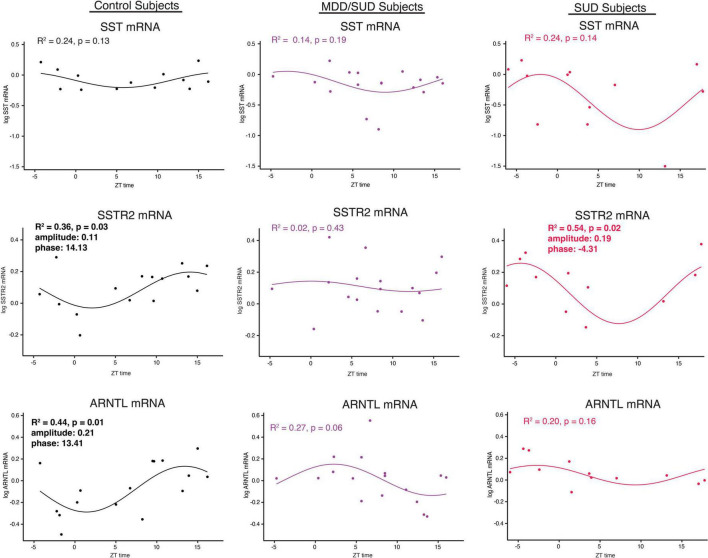
Diurnal rhythms of SST Signaling and ARNTL gene expression. Diurnal gene expression rhythms for Sst signaling genes and Arntl demonstrating enhanced rhythms in subjects with SUD. Expression level values in the scatterplots are indicated on the y axis with each dot representing an individual subject and the x axis representing ZT time of death values. The colored lines represent the fit sinusoidal curve for each marker in each diagnosis group. *R*^2^ values and corresponding *p*-values represent significance of fit for diurnal expression rhythms. Significant rhythms were observed in control subjects for Sstr2 and ARNTL expression, and enhanced rhythm and altered phase was detected for Sstr2 in subjects with SUD while Arntl rhythms were lost in subjects with SUD and MDD/SUD. Phase and amplitude estimates and *p*-values are indicated on each scatterplot for outcome measures with significant *R*^2^ values.

Non-linear regression analysis revealed weak, non-significant rhythms for Sst in control subjects and subjects with SUD or MDD/SUD (Figures 6A,B). A significant rhythm was observed for Sstr2 expression in control subjects, with a phase estimate of 14.13 (Figure 6D). Subjects with SUD also displayed a significant rhythm in Sstr2 expression (Figure 6F), with a phase estimate of –4.31. The difference in phase of Sstr2 expression rhythm between control subjects and subjects with SUD also was statistically significant (t-value: –9.56, *p* < 0.0001). Subjects with MDD/SUD had no significant rhythm in Sstr2 expression (Figure 6E).

**FIGURE 7:**
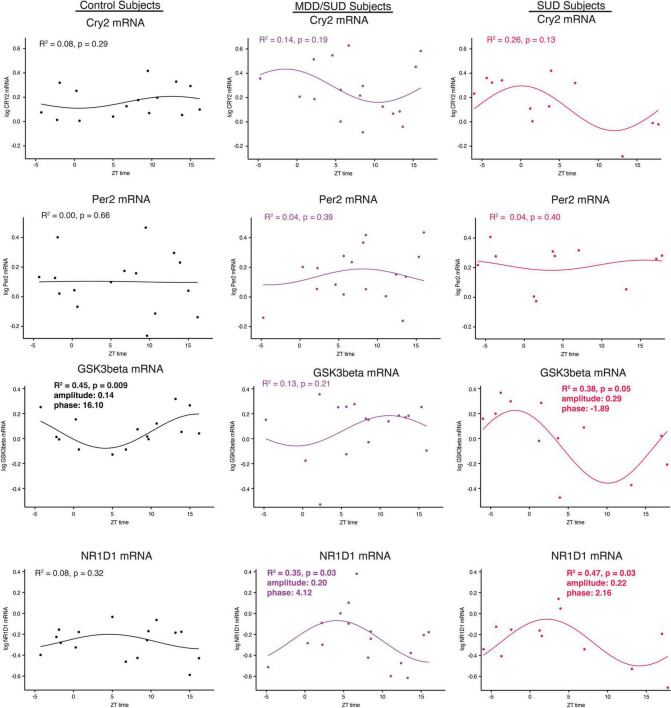
Diurnal clock gene expression rhythms. Diurnal gene expression rhythms for clock genes in the human hippocampus. Expression level values in the scatterplots are indicated on the y axis with each dot representing an individual subject and the x axis representing ZT time of death values. The colored lines represent the fit sinusoidal curve for each marker in each diagnosis group. *R*^2^ values and corresponding *p*-values represent significance of fit for diurnal expression rhythms. Significant diurnal expression rhythm was observed for Gsk3β in control subjects. Enhanced rhythms were observed in subjects with SUD for Gsk3β and Nr1d1 gene expression and for Nr1d1 in subjects with MDD/SUD. Phase and amplitude estimates and *p*-values are indicated on each scatterplot for outcome measures with significant *R*^2^ values.

Arntl gene expression was significantly rhythmic in control subjects (Figure 6H), with a phase of 13.41. Significant rhythmicity for Arntl was not detected in subjects with SUD or subjects with MDD/SUD (Figures 6I,J). No significant gene expression rhythms were detected for Cry2 or Per2 in any diagnosis group (Figures 7A–F). Significant rhythmicity was observed for Gsk3β in control subjects (Figure 7G) with a phase of 16.10. Subjects with SUD had a significant rhythm of Gsk3β expression with a phase of –1.89 (Figure 7I). The phase difference between subjects with SUD and control subjects was also significant (t-value: –9.87, *p* < 0.0001). Lastly, Nr1d1 expression was not significantly rhythmic in control subjects (Figure 7J). However, subjects with MDD/SUD and subjects with SUD had significant rhythms in Nr1d1 expression, with phases of 4.12 and 2.16, respectively (Figures 7K,L).

## Discussion

**FIGURE 8:**
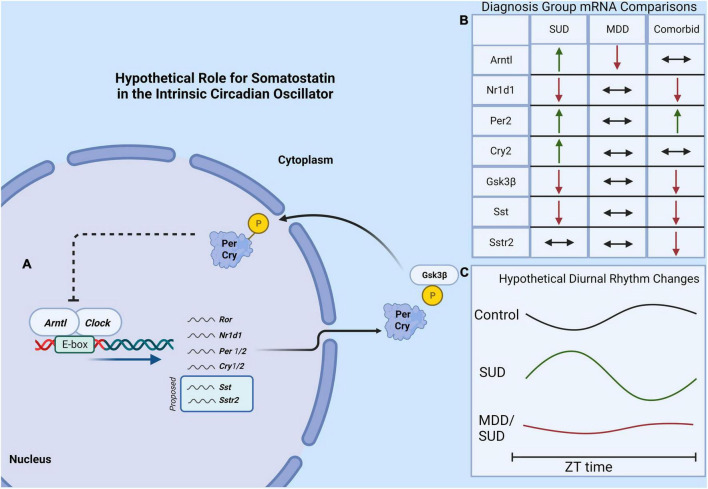
Hypothetical model of molecular clock and SST signaling in SUD. **(A)** The core molecular circadian clock depicting the dimerization of Arntl and Clock genes which promotes the expression of several “clock-controlled genes” which may potentially include Sst and the Sstr2. **(B)** A summary of findings of diagnosis group differences in gene expression. **(C)** A hypothetical model showing altered clock gene rhythms in subjects with SUD and MDD/SUD, with amplified and shifted diurnal rhythms in SUD compared to dampened rhythms in the MDD/SUD group.

Our results provide the first evidence for altered SST signaling and clock gene expression in the hippocampus of subjects with SUD and add to the growing evidence for decreased SST signaling and clock genes in MDD (Figure 8). The observed effects of substances in the blood at death including alcohol on SST and SSTR2 and cocaine and opioids on clock genes point to the importance for including information from toxicology reports in analysis of human postmortem brain tissue from subjects with SUD. Furthermore, the effects of ZT on our measures are in line with recent studies from other groups demonstrating the importance of including ZT in analysis of human postmortem brain samples, as variability due to ZT may mask expression differences between diagnosis groups that occur primarily during the night or during the day (Seney et al., 2019, 2021). Our study represents a first step in testing SST signaling and molecular clock abnormalities in the brain of subjects with SUD and comorbid disorders. Although this study is focused on MDD as a disorder often comorbid with SUD, several other disorders also display comorbidity with SUD including anxiety disorders, bipolar disorder, and schizophrenia. Our cohort includes subjects with anxiety disorders, obsessive compulsive disorder, and personality disorders. No effects of presence or absence of these disorders were detected in our ANCOVA models. Ongoing studies testing this relationship of molecular pathology in SUD in subjects with comorbid bipolar disorder or subjects with schizophrenia will examine comorbidity with other disorders.

### Sleep disturbances in substance use disorders and major depressive disorder subjects

Subjects with SUD and MDD/SUD in our cohort had significantly higher prevalence of sleep disturbances (Figure 5) in line with extensive reports of sleep disturbances in these disorders (Brower et al., 1998, 2001; Brower and Hall, 2001; Mahfoud et al., 2009; Baglioni et al., 2011; Canellas and de Lecea, 2012; Irwin et al., 2022). This prevalence of sleep disturbances suggests that our cohort reflects the real-world population of people with SUD and/or MDD. Our results regarding SST signaling and clock gene expression will be considered in the context of subjects with sleep disturbances.

### Altered somatostatin and SSTR2 mRNA expression in subjects with substance use disorders

Our data suggest SST expression is decreased in subjects with SUD, and acute alcohol exposure (as indicated by ethanol in blood at time of death) increases both SST and SSTR2 expression (Figure 1), potentially compensating for decreased SST signaling. Alternatively, chronic alcohol use may result in a compensatory decrease in SST levels, however, the lack of correlation with Sst and Sstr2 mRNA expression with duration of alcohol use (Supplementary Figure 1) suggests that this is not the case. The observed effect of increased Sstr2 expression in subjects with alcohol in the blood at death is in line with preclinical studies reporting increased SSTR expression in the rat hippocampus following acute exposure to both alcohol and cocaine (Barrios et al., 1990). Hippocampal SSTR4 stimulation also enhances contextual memory (Gastambide et al., 2009), suggesting that hippocampal SST signaling is upregulated during contextual learning. Systemic alcohol administration in rats increases both amplitude and duration of postsynaptic inhibitory responses to somatostatin, providing further support for enhanced hippocampal SST signaling following acute alcohol exposure (Mancillas et al., 1986).

Preclinical studies support a key role for SST signaling in reward processing (Yuferov et al., 2003; Stengel et al., 2010a; Jonsson et al., 2014; Kim et al., 2017). For example, intracerebroventricular administration of SST agonists in mice and rats during the light cycle promotes food intake during the normal resting period (Stengel et al., 2010b,c). Furthermore, this effect was partially dependent on mu-opioid receptors (Stengel et al., 2010a). Growing evidence points to a critical role of SST signaling in encoding and regulation of memories (Kluge et al., 2008; Delorme et al., 2021; Morales et al., 2021). For example, SST neurons in the dentate gyrus are necessary for pattern separation during contextual memory encoding, and inhibition of dentate gyrus SST neurons results in impaired discrimination of contextual memories (Morales et al., 2021). SST knockout or depletion also results in decreased LTP in CA1 hippocampus (Kluge et al., 2008). In comparison, decreased activity of SST neurons onto hippocampal dentate gyrus cells is important for consolidation of contextual memory (Delorme et al., 2021). Recent work demonstrates that sleep deprivation activates hippocampal SST neurons which in turn interferes with sleep-dependent consolidation of contextual memories (Delorme et al., 2021). SSTR2 signaling contributes to fear memory processing (Engin et al., 2008; Engin and Treit, 2009; Yeung and Treit, 2012), and SSTR2 and 4 have been reported to contribute to reward/feeding (Yuferov et al., 2003; Stengel et al., 2010a; Jonsson et al., 2014; Kim et al., 2017). Speculatively, our findings regarding SSTR2 expression and sleep disturbances in subjects with SUD may contribute to impaired consolidation of contextual reward memories that can promote relapse.

### Decreased Sst and Sstr2 mRNA expression in subjects with major depressive disorder

Decreased Sst and Sstr2 mRNA in subjects with MDD are in line with a growing number of reports for decreased SST signaling in MDD (Viollet et al., 2000; Lin and Sibille, 2015; Prevot et al., 2017; Sibille, 2017). Decreased SST signaling may be involved in anhedonia inherent to MDD (Viollet et al., 2000; Lin and Sibille, 2015; Prevot et al., 2017; Sibille, 2017). Preclinical findings support this notion. SSTR2 KO mice display increased depression-like and anxiety-like behaviors together with increased cortisol levels (Viollet et al., 2000; Prevot et al., 2017). Conversely, injection of SSTR2 agonist in the rodent hippocampus decreases depression-like and anxiety-like behaviors (Prevot et al., 2017). Speculatively, our data suggest that substance use including alcohol may partially compensate for decreased SST signaling in MDD, as reflected by the lack of changes in Sst and Sstr2 expression in subjects with MDD/SUD.

### Altered clock gene expression in subjects with substance use disorders

We observed significant increases in expression of several clock genes in subjects with SUD. The increased clock gene expression is similar to increased clock gene expression in the rat hippocampus following chronic cocaine exposure (Wang et al., 2019) and is in line with a recent report of enhanced molecular rhythms in several signaling pathways in subjects with opioid use disorder (Seney et al., 2021). Furthermore, the enhanced diurnal rhythms observed in subjects with SUD of Gsk3β and Nr1d1 and Sstr2 expression in our study (Figures 6, 7) is in line with these preclinical and postmortem studies (Wang et al., 2019; Seney et al., 2021). Increased clock gene expression in the hippocampus of subjects with SUD suggests environmental signals such as exposure to rewarding substances may entrain and enhance molecular clock gene rhythms. Rodent studies showing entrainment of clock gene rhythms in several brain areas including the hippocampus in response to restricted feeding or fear exposure support this hypothesis (Verwey et al., 2007; Pantazopoulos et al., 2011). Clock gene rhythms may synchronize to administration of rewarding substances but may result in misalignment of biological rhythms with the environment. Misalignment of biological rhythms with the environment due to disrupted circadian rhythms from chronic drug use is proposed as a major contributing factor to relapse (Tamura et al., 2021). Non-linear regression analysis of clock gene expression rhythms in our study suggesting altered phases in subjects with SUD and MDD/SUD compared to control subjects provides further support that hippocampal expression rhythms in these subjects may be synchronized by rewarding drug stimuli resulting in misalignment with the environment (Figure 7).

In our study, subjects with SUD displayed decreased Gsk3β expression and increased Arntl expression, suggesting that the decrease in Gsk3β may contribute to our observed increase in Arntl increased in SUD. We also observed enhanced Nr1d1 and Gsk3β diurnal rhythms together with loss of Arntl rhythm in subjects with SUD (Figures 6, 7). The enhanced rhythm of Gsk3β in SUD was due largely to a decrease of Gsk3β expression in the second half of the ZT scale. Furthermore, the phase of Gsk3β diurnal rhythms was also significantly different between subjects with SUD and control subjects. GSK3β stabilizes NR1D1 through phosphorylation of NR1D1, and inhibition of GSK3β results in NR1D1 degradation and in turn increased activity of ARNTL as NR1D1 normally represses ARNTL (Yin et al., 2006). Increased Arntl expression together with dampened diurnal expression rhythm of Arntl suggests that the molecular clock is locked in the “on” position in SUD, possibly through reduced GSK3β resulting in NR1D1 degradation and in turn increased ARNTL. Increased expression of Per2 and Cry2 in subjects with SUD supports this hypothesis. Persistent increased Arntl expression may potentially influence expression of a broad range of clock-controlled genes. However, the observed increase in Nr1d1 expression does not support this scenario, but this may be due to effects of cocaine in the blood at death on Nr1d1 expression in our ANCOVA model that may impact this interpretation. Protein phosphorylation is a major factor on regulation of the molecular clock. GSK3β kinase phosphorylates ARNTL and promotes ARNTL ubiquitination (Sahar et al., 2010). Lack of this GSK3β phosphorylation dampens circadian gene expression of ARNTL (Sahar et al., 2010). In turn, GSK3β activity is inhibited by GSK3β phosphorylation (Fang et al., 2000). Furthermore, subcellular localization of molecules including clock molecules and SST signaling molecules is critically involved in their functions. Future studies with greater sampling across the ZT scale and on proteomics and kinomics of clock molecules in the hippocampus of subjects with SUD may provide insight into how the molecular clock is altered in this region in subjects with SUD.

Several studies suggest that altered clock gene expression can impact drug-taking behaviors. Pre-clinical evidence using rodents carrying deletions of molecular clock genes have found variations in drug-taking behavior. For example, mice with a mutation in the Clock gene display increased ethanol and cocaine intake (McClung et al., 2005; Ozburn et al., 2013). In addition, mice carrying deletions of Npas2, a homolog of Clock with preferential expression in the striatum and nucleus accumbens (NAc), display increased cocaine-seeking and self-administration behavior (DePoy et al., 2021). Recent evidence from the same group has identified altered diurnal rhythms of dopamine, opioid, and GABAergic synaptic signaling in the NAc and dorsolateral prefrontal cortex in human subjects with opioid use disorder (Xue et al., 2021).

Altered clock gene expression in the hippocampus of subjects with SUD also may impact reward memory processing. Clock genes in the hippocampus regulate diurnal rhythms of encoding and retrieval of contextual memories (Snider et al., 2018). Genetic inhibition of Arntl in the mouse hippocampus disrupts dopamine dependent retrieval of hippocampal memories between ZT8-12 (Hasegawa et al., 2019). Furthermore, dopamine signaling from the VTA is involved in enhancing hippocampal encoding during reward learning (Murty and Adcock, 2014). Our observed changes in Arntl expression in subjects with SUD may contribute to regulation of contextual reward memory in the hippocampus modulated by dopamine signaling. Genetic polymorphisms for Arntl have been associated with alcohol consumption, increased risk of alcohol use disorder, and alcohol use disorder with comorbid MDD or bipolar disorder (Kovanen et al., 2010; Sjoholm et al., 2010; Partonen, 2015; Banach et al., 2018), indicating increased ARNTL expression may reflect core genetic factors associated with SUD. Similar increases in alcohol intake were reported in mice with genetic mutations in Per1 or Per2 genes (Gamsby et al., 2013), and a genetic polymorphism for Per2 was associated with both sleep disturbances and increased alcohol consumption (Comasco et al., 2010), suggesting that sleep disturbances and substance use disorders may arise from shared genetic factors.

### Altered clock gene expression in subjects with major depressive disorder

Several lines of evidence point to sleep and circadian rhythm disturbances as key components of mood disorders including MDD. Sleep disturbances are described as a core feature of MDD (Baglioni et al., 2011; Irwin et al., 2022). Altered rhythms in several circadian rhythm regulated systemic factors are also reported in subjects with MDD or bipolar disorder including blood pressure, melatonin and cortisol (Atkinson et al., 1975; Kennedy et al., 1989; Souetre et al., 1989; Yehuda et al., 1996; Cervantes et al., 2001; Keller et al., 2006; Moon et al., 2016). Furthermore, clock genes including Per1 and Per2 were identified as common network molecular factors in preclinical models of sleep loss and MDD (Scarpa et al., 2018). Polymorphisms in clock genes have also been associated with MDD including Cry1 and Npas2 (Soria et al., 2010) and a Clock gene polymorphism was associated with insomnia and relapse in people with MDD (Serretti et al., 2004, 2005).

Several preclinical models of depression report altered clock gene rhythms in several brain areas. Sleep and circadian rhythm disturbances in mice following chronic social defeat are accompanied by altered clock gene expression in several brain regions (Wells et al., 2017). Similarly, unpredictable chronic mild stress results in region specific changes in clock gene expression including decreased amplitude of molecular clock rhythms in the SCN, increased rhythmicity in the nucleus accumbens (Logan et al., 2015; Christiansen et al., 2016), and reduced nuclear expression of Arntl and Clock protein in the prefrontal cortex (Calabrese et al., 2016). Notably, exposing rats to chronic unpredictable mild stressors induces a shift in clock gene rhythms including ARNTL in the hippocampus, and viral vector knockdown of Clock expression in the hippocampus induces depressive-like behavior (Jiang et al., 2013).

Our observed decrease of Arntl expression in the hippocampus of subjects with MDD is in line with reports of weaker molecular clock rhythms in subjects with MDD (Li et al., 2013), and rodent studies suggest weaker rhythms in depression may result from decreased Arntl expression in the suprachiasmatic nucleus (Landgraf et al., 2016; Vadnie et al., 2021). Decreased hippocampal Arntl expression impairs hippocampal dependent memory including contextual fear and spatial memory and hippocampal LTP (Wardlaw et al., 2014). Our observed decrease in Arntl expression in the hippocampus of subjects with MDD may impair a wide range of circadian processes in the hippocampus that regulate memory processing (Snider et al., 2018). Decreased clock gene expression also results in impaired hippocampal neurogenesis (Bouchard-Cannon et al., 2013; Malik et al., 2015). Impaired hippocampal neurogenesis has been proposed as a core concept of MDD (Kempermann and Kronenberg, 2003; Sahay and Hen, 2007; Lee et al., 2013). Impaired hippocampal neurogenesis can contribute to contextual memory dysfunction through impairing pattern separation due to decreased resolution of contextual memories (Clelland et al., 2009). Together with the observed decrease in Sst and Sstr2 expression in the same subjects and evidence that hippocampal SST signaling regulates memory consolidation during sleep (Delorme et al., 2021), this points to two processes that may converge to promote impaired memory processing in MDD. Sleep disturbances, commonly reported by people suffering from MDD (Baglioni et al., 2011; Irwin et al., 2022), may in turn contribute to decreased expression of hippocampal ARNTL and SST and impaired memory consolidation.

In summary, our results point to a key role for SST signaling in the hippocampus of subjects with SUD, together with sleep disturbances and changes in clock gene expression, which may potentially impact context-induced relapse. In comparison, our data in subjects with MDD add to the growing evidence for impaired SST signaling and molecular clock rhythms in this disorder. Our study consisted of whole hippocampus mRNA expression analysis. Future high resolution microscopy studies will define the cell type and subregion specific neurocircuitry behind these findings. Our ZT diurnal expression rhythm analysis represents a first step toward examining potential alterations in hippocampal molecular diurnal rhythms in SUD and MDD. Future studies with greater ZT sampling evenly balanced between diagnosis groups will shed light into diurnal hippocampal molecular rhythms in SUD and MDD.

## Data availability statement

The original contributions presented in this study are included in the article/Supplementary material, further inquiries can be directed to the corresponding author.

## Author contributions

HP designed the studies, analyzed data, and wrote the manuscript. JV contributed to study design, collected data, analyzed data, and wrote the manuscript. SO’D, BG, DP, and CS contributed to study design, data collection, and manuscript preparation. DS and RB contributed to data collection. WW contributed to study design, data analysis, and manuscript preparation. All authors contributed to the article and approved the submitted version.

## Supplementary material

Supplementary Figure 1 Duration of alcohol use disorder is not correlated with expression of SST or SSTR2. Duration of alcohol use disorder was not correlated with SST expression in subjects with SUD **(A)** or MDD/SUD **(B)**. Similarly, no correlation was observed for duration of alcohol use disorder with expression of SSTR2 in subjects with SUD **(C)** or subjects with MDD/SUD **(D)**.
