# Supplementary material for: Altered expression of somatostatin signaling molecules and clock genes in the hippocampus of subjects with substance use disorder
Source: Front Neurosci. 2022 Sep 7;16:903941. doi: 10.3389/fnins.2022.903941 (PMC9489843; doi:10.3389/fnins.2022.903941)
Supplement: Supplementary file 1 [file Data_Sheet_1.PDF]

Tables 1-4: Basic demographic information for all diagnosis groups

Table 1: Basic demographic information for subjects with substance use disorder

Age	Sex	Race	pH	PMI (hrs)	Sleep quality	ZT time
			**SUBSTANCE USE DISORDER**			
40	M	Black	6.5	7	*NA*	-2.42
32	M	Black	6.82	14	*NA*	-6
29	M	White	6.75	27	*NA*	3.92
30	M	White	6.49	12	*NA*	-3.7
38	M	White	6.68	25	Decreased sleep	7.02
38	M	White	6.42	6	*NA*	*NA*
49	M	White	6.88	24	*NA*	*NA*
34	M	Black	6.5	16	*NA*	17.77
36	M	Black	6.97	38.5	Decreased sleep	-4.37
30	M	Black	6.8	21	*NA*	*NA*
42	M	White	6.86	30	*NA*	1.23
38	M	White	6.62	12	*NA*	17.03
31	F	White	6.18	12	Decreased sleep	*NA*
50	M	White	6.52	24	*NA*	*NA*
35	F	Black	6.43	26	*NA*	*NA*
22	F	White	6.12	12	*NA*	*NA*
56	M	Black	6.51	17	*NA*	13.14
28	M	White	6.3	26	Decreased sleep	3.67
52	M	White	6.81	8	*NA*	1.53
24	M	White	6.28	14	*NA*	*NA*
mean ± SD 34.8±9.0	3F, 17M	7B, 13W	6.57±0.23	18.58±8.5		

Table 2: Basic demographic information for subjects with major depressive disorder

Age	Sex	Race	pH	PMI (hrs)	Sleep quality	ZT time
**MAJOR DEPRESSIVE DISORDER**						
43	M	White	6.73	21	Decreased sleep	6.25
63	F	White	6.3	18	Decreased sleep	10.53
42	M	White	6.64	20	*NA*	*NA*
50	F	White	6.83	23	Decreased sleep	6
34	F	White	6.27	24	Decreased sleep	NA
55	M	White	6.64	29	Decreased sleep	2.08
33	M	White	6.79	18	Increased sleep	*NA*
53	M	White	6.73	29	Decreased sleep	4.25
44	F	White	6.71	29	*NA*	2.83
59	M	White	6.22	27	Decreased sleep	8.47
46	M	Black	6.26	17	Decreased sleep	13.47
36	F	White	6.84	25	Increased sleep	7.12
35	M	White	6.96	11	Decreased sleep	11.33
41	M	White	6.24	19	Increased sleep	*NA*
56	F	White	5.61	38	Decreased sleep	*NA*
62	M	Black	6.06	22	Increased sleep	*NA*
20	M	White	6.73	20	Decreased sleep	*NA*
59	M	Black	6.6	31	Decreased sleep	*NA*
48	M	White	6.41	27	Normal sleep	2.78
61	M	White	6.74	25	Decreased sleep	6.33
mean ± SD 47±11.4	6F, 14M	3B, 17W	6.5±0.32	23.7±5.9		

Table 3: Basic demographic information for subjects with comorbid substance use disorder and major depressive disorder

Age	Sex	Race	pH	PMI (hrs)	Sleep quality	ZT time
**COMORBID SUD AND MDD**						
36	M	White	6.72	15	Decreased sleep	15.33
37	M	White	6.89	19	*NA*	11.08
41	F	White	6.55	17	Decreased sleep	*NA*
47	F	White	6.65	9	*NA*	-3.5
48	F	White	6.13	24	Increased sleep	2.25
44	M	White	6.77	20	Decreased sleep	8.16
29	F	White	6.47	29	Decreased sleep	4.58
59	F	White	6.8	24	*NA*	6.68
40	M	White	6.66	26	*NA*	2.16
45	M	White	6.29	24	*NA*	*NA*
20	M	Black	6.21	10	Increased sleep	*NA*
35	M	White	6.81	24	*NA*	5.62
34	M	White	6.33	17	*NA*	13.25
37	M	White	6.59	18	*NA*	14
63	F	White	6.32	24	Decreased sleep	5.63
62	M	Black	6.52	17	Decreased sleep	15.98
54	M	White	6.54	38	Decreased sleep	8.5
48	F	Black	5.87	17	Decreased sleep	*NA*
43	M	White	6.6	20	Decreased sleep	13.65
42	F	White	6.84	12	Decreased sleep	-4.75
40	M	White	6.62	21	Normal sleep	8.5
58	M	Black	6.11	37	*NA*	*NA*
30	M	White	6.91	18	*NA*	12.42
62	M	White	6.7	5	*NA*	0.37
mean ± SD 43.9±11.1	8F, 16M	4B, 20W	6.54±0.27	20.2±7.6		

Table 4: Basic demographic information for unaffected control subjects

Age	Sex	Race	pH	PMI (hrs)	Sleep quality	ZT time
			**UNAFFECTED CONTROLS**			
62	F	White	6.34	27.5	Normal sleep	0.67
54	M	Black	6.53	19	Normal sleep	9.67
52	M	White	6.28	17	Normal sleep	13.15
30	M	Black	6.98	19	Normal sleep	13.92
48	M	Black	6.98	9	Normal sleep	0.28
51	F	Black	6.3	22	Normal sleep	8.25
49	F	Black	6.57	29	Normal sleep	5
38	F	White	5.93	13	Normal sleep	-1.88
44	F	Black	6.72	32	Decreased sleep	2.17
44	M	White	6.6	24.32	Normal sleep	*NA*
28	M	Black	6.32	35.3	Normal sleep	-2.22
42	F	White	6.6	21	Normal sleep	10.72
31	M	White	6.78	14.15	Normal sleep	15
51	M	White	6.76	17	Decreased sleep	16.17
29	F	Black	6.64	25.5	Normal sleep	*NA*
35	M	Black	6.26	21	Normal sleep	9.5
49	M	White	6.71	9.75	Decreased sleep	-1.67
17	M	White	6.66	22.75	Normal sleep	-4.27
51	M	White	6.3		Normal sleep	*NA*
59	M	White	6.47	23.75	Normal sleep	6.75
mean ± SD 43.2±11.42	7F, 13M	9B, 11W	6.5±0.25	21.2±6.8		

Tables 5-8: Substance use information for all diagnostic groups

Table 6: Substance use information for subjects with substance use disorder

case	Onset of AUD (age)	Duration of AUD (yrs)	Ethanol in toxicology report	Cocaine in toxicology report	Opioids in toxicology report	Opioid dependence	Smoker	Nicotine rating	Alcohol rating	Cannabis history	Drug abuse type
**SUBSTANCE USE DISORDER**											
40M	14	26	Yes	Yes	No	No	Yes	2	4	No	Polysubstance abuse
32M	*NA*	*NA*	Yes	Yes	No	No	Yes	3	2	Yes	Polysubstance abuse
29M	14	15	No	No	No	No	Yes	4	4	Yes	Polysubstance abuse
30M	20	10	Yes	No	No	No	Yes	*NA*	4	Yes	Polysubstance abuse
38M	20	18	No	No	Yes	Yes	No	0	2	Yes	Polysubstance abuse
38M	16	22	Yes	No	Yes	Yes	Yes	3	4	Yes	Polysubstance abuse
49M	18	31	Yes	No	No	Yes	Yes	4	4	Yes	Polysubstance abuse
34M	16	18	No	Yes	No	No	Yes	4	4	Yes	Polysubstance abuse
36M	16	20	Yes	No	No	No	Yes	2	4	Yes	Polysubstance abuse
30M	19	11	No	Yes	No	No	Yes	2	3	Yes	Polysubstance abuse
42M	15	27	No	No	No	No	Yes	4	4	No	Alcohol abuse
38M	14	24	Yes	Yes	Yes	Yes	Yes	4	4	Yes	Polysubstance abuse
31F	16	15	No	No	No	No	Yes	4	4	Yes	Polysubstance abuse
50M	14	36	Yes	Yes	No	Yes	No	0	4	No	Polysubstance abuse
35F	15	20	No	Yes	No	No	No	0	4	Yes	Polysubstance abuse
22F	12	10	Yes	No	No	No	Yes	4	4	Yes	Polysubstance abuse
56M	26	30	No	Yes	No	No	No	0	4	Yes	Polysubstance abuse
28M	*NA*	*NA*	No	No	Yes	Yes	Yes	2	4	Yes	Polysubstance abuse
52M	16	35	Yes	No	No	No	Yes	4	4	Yes	Polysubstance abuse
24M	21	3	Yes	Yes	Yes	Yes	Yes	4	4	Yes	Polysubstance abuse
mean ± SD 34.8±9.0/ 3F, 17M											

Table 7: Substance use information for subjects with major depressive disorder

case	Onset of AUD (age)	Duration of AUD (yrs)	Ethanol in toxicology report	Cocaine in toxicology report	Opioids in toxicology report	Opioid dependence	Smoker	Nicotine rating	Alcohol rating	Cannabis history	Drug abuse type
**MAJOR DEPRESSIVE DISORDER**											
43M	None	0	No	No	No	No	No	0	0	No	None
63F	None	0	No	No	No	No	Yes	4	1	No	None
42M	None	0	No	No	No	No	No	0	0	No	None
50F	None	0	No	No	No	No	Yes	4	1	No	None
34F	None	0	No	No	No	No	No	0	0	No	None
55M	None	0	No	No	No	No	Yes	3	0	No	None
33M	None	0	No	No	No	No	No	0	1	No	None
53M	None	0	No	No	No	No	No	0	1	No	None
44F	None	0	No	No	No	No	Yes	3	0	No	None
59M	None	0	No	No	No	No	Yes	4	2	No	None
46M	None	0	No	No	No	No	No	0	2	No	None
36F	None	0	No	No	No	No	Yes	4	2	No	None
35M	None	0	No	No	No	No	No	0	1	No	None
41M	None	0	No	No	No	No	Yes	4	1	No	None
56F	None	0	No	No	No	No	Yes	4	2	No	None
62M	None	0	No	No	No	No	Yes	3	1	No	None
20M	None	0	No	No	No	No	No	0	1	No	None
59M	None	0	No	No	No	No	No	0	1	No	None
48M	None	0	No	No	No	No	Yes	3	1	No	None
61M	None	0	No	No	Yes	No	Yes	2	1	No	None
mean ± SD 47±11.4/ 6F, 14M											

Table 8: Substance use information for subjects with comorbid substance use disorder and major depressive disorder

case	Onset of AUD (age)	Duration of AUD (yrs)	Ethanol in toxicology report	Cocaine in toxicology report	Opioids in toxicology report	Opioid dependence	Smoker	Nicotine rating	Alcohol rating	Cannabis history	Drug abuse type
**COMORBID SUD AND MDD**											
36M	8	28	Yes	Yes	No	No	Yes	4	4	Yes	Polysubstance abuse
37M	14	23	Yes	No	No	No	Yes	4	4	Yes	Polysubstance abuse
41F	*NA*	*NA*	Yes	No	No	Yes	No	*NA*	1	No	Polysubstance abuse
47F	27	20	No	No	No	Yes	Yes	4	4	Yes	Polysubstance abuse
48F	45	3	Yes	No	No	No	Yes	4	4	No	Polysubstance abuse
44M	34	10	No	No	No	No	Yes	4	4	No	Polysubstance abuse
29F	*NA*	*NA*	No	No	Yes	Yes	Yes	4	4	No	Polysubstance abuse
59F	34	25	No	No	Yes	No	Yes	4	4	No	Polysubstance abuse
40M	13	27	Yes	Yes	No	No	Yes	*NA*	4	Yes	Polysubstance abuse
45M	*NA*	*NA*	Yes	No	No	No	Yes	4	4	No	Polysubstance abuse
20M	19	1	No	No	No	No	No	0	3	Yes	Polysubstance abuse
35M	*NA*	*NA*	No	No	No	No	No	0	4	No	Polysubstance abuse
34M	14	20	Yes	No	No	No	Yes	2	3	Yes	Polysubstance abuse
37M	14	23	Yes	No	Yes	Yes	Yes	3	4	No	Polysubstance abuse
63F	38	25	Yes	No	No	Yes	Yes	4	4	No	Polysubstance abuse
62M	42	20	Yes	No	No	No	Yes	2	4	No	Polysubstance abuse
54M	24	30	No	No	No	No	Yes	4	4	No	Polysubstance abuse
48F	*NA*	*NA*	Yes	No	Yes	Yes	Yes	3	4	Yes	Polysubstance abuse
43M	*NA*	*NA*	Yes	No	No	Yes	Yes	4	1	No	Polysubstance abuse
42F	*NA*	*NA*	No	No	No	Yes	Yes	1	3	Yes	Polysubstance abuse
40M	*NA*	*NA*	No	No	No	No	Yes	4	2	Yes	Polysubstance abuse
58M	23	35	Yes	No	Yes	Yes	Yes	4	4	No	Polysubstance abuse
30M	*NA*	*NA*	Yes	No	No	No	Yes	3	3	Yes	Alcohol abuse
62M	*NA*	20	No	No	No	No	Yes	4	4	No	*NA*
mean ± SD 43.9±11.1/ 8F, 16M											

Table 9: Substance use information for unaffected control subjects

case	Onset of AUD (age)	Duration of AUD (yrs)	Ethanol in toxicology report	Cocaine in toxicology report	Opioids in toxicology report	Opioid dependence	Smoker	Nicotine rating	Alcohol rating	Cannabis history	Drug abuse type
**UNAFFECTED CONTROLS**											
62F	None	0	No	No	No	No	No	0	1	No	None
54M	None	0	No	No	No	No	No	4	3	No	None
52M	None	0	No	No	No	No	No	0	0	No	None
30M	None	0	No	No	No	No	No	1	1	No	None
48M	None	0	No	No	No	No	No	4	1	No	None
51F	None	0	No	No	No	No	Yes	4	1	Yes	Cannabis
49F	None	0	No	No	No	No	No	0	3	No	None
38F	None	0	No	No	No	No	No	0	0	No	None
44F	None	0	No	No	No	No	Yes	2	1	No	None
44M	None	0	No	No	No	No	Yes	4	1	No	None
28M	None	0	No	No	No	No	No	0	1	No	None
42F	None	0	No	No	No	No	Yes	4	2	Yes	Cannabis
31M	None	0	No	No	No	No	Yes	4	2	No	None
51M	None	0	No	No	No	No	Yes	4	2	No	None
29F	None	0	No	No	No	No	Yes	2	2	Yes	Cannabis
35M	None	0	No	No	No	No	Yes	2	2	No	None
49M	None	0	No	No	No	No	Yes	4	2	Yes	Cannabis
17M	None	0	Yes	No	No	No	No	0	2	No	None
51M	None	0	No	No	No	No	No	1	2	Yes	Cannabis
59M	None	0	No	No	No	No	Yes	3	1	No	None
mean ± SD 43.2±11.42/ 7F, 13M											

Tables 9-12: Mood disorder-related information for all diagnostic groups

Table 9: Mood disorder-related information for subjects with substance use disorders

case	Suicide	Depression severity	Duration of MDD (yrs.)	Antidepressants in blood at death	Antidepressants/ month (grams)	Mania	Psychosis	Antipsychotics
**SUBSTANCE USE DISORDERS**								
62F	No	None	0	No	0	No	No	No
54M	No	None	0	No	0	No	No	No
52M	No	Mild	0	No	0	No	No	No
30M	No	None	0	No	0	No	No	No
48M	No	*NA*	0	Yes	4.5	No	No	No
51F	No	None	0	No	0	No	No	No
49F	Yes	None	0	No	0	No	No	No
38F	No	None	0	No	0	No	No	No
44F	No	None	0	No	0	No	No	No
44M	No	None	0	No	0	No	No	No
28M	No	None	0	No	0	No	No	No
42F	No	None	0	No	0	No	No	No
31M	No	*NA*	*NA*	Yes	10.2	No	Yes	Yes
51M	Yes	*NA*	*NA*	Yes	4.8	No	No	No
29F	No	None	*NA*	No	0	No	No	No
35M	No	None	0	No	0	No	No	No
49M	No	None	0	No	0	No	No	No
17M	No	None	0	No	0	No	No	No
51M	No	*NA*	*NA*	No	0	No	No	No
59M	Yes	*NA*	*NA*	Yes	2.6	No	No	No
mean ± SD 34.8±9.0/ 3F, 17M								

Table 10: Mood disorder-related information for subjects with major depressive disorder

case	Suicide	Depression severity	Duration of MDD (yrs.)	Antidepressants in blood at death	Antidepressants/ month (grams)	Mania	Psychosis	Antipsychotics
**MAJOR DEPRESSIVE DISORDER**								
43M	Yes	Moderate	15	No	0	No	Yes	No
63F	No	Moderate	*NA*	Yes	3	No	No	No
42M	Yes	Severe	*NA*	Yes	0.8	No	No	No
50F	Yes	Moderate	*NA*	Yes	0	No	Yes	No
34F	Yes	Severe	*NA*	Yes	3	No	No	Yes
55M	Yes	*NA*	*NA*	Yes	3.2	No	No	No
33M	Yes	Moderate	*NA*	Yes	16.7	No	No	Yes
53M	Yes	*NA*	*NA*	Yes	4	No	No	No
44F	Yes	*NA*	12	Yes	8.7	No	No	No
59M	Yes	*NA*	0.08	Yes	3	No	No	No
46M	No	Mild	1	No	0	No	No	No
36F	No	Mild	*NA*	No	0	No	No	No
35M	No	Moderate	1.5	No	0	No	No	No
41M	No	Moderate	7	No	0	No	No	No
56F	No	*NA*	1	No	0	No	No	No
62M	No	Mild	49	No	0	No	No	No
20M	Yes	Moderate	1.33	No	0	No	No	No
59M	Yes	*NA*	*NA*	Yes	0.4	No	No	No
48M	No	*NA*	*NA*	Yes	2.7	No	No	No
61M	Yes	Severe	47	Yes	3.6	No	No	No
mean ± SD 47±11.4/ 6F, 14M								

Table 11: Mood disorder-related information for subjects with comorbid substance use disorder and major depressive disorder

case	Suicide	Depression severity	Duration of MDD (yrs.)	Antidepressants in blood at death	Antidepressants/ month (grams)	Mania	Psychosis	Antipsychotics
				**COMORBID SUD AND MDD**				
36M	Yes	Severe	16	Yes	3	No	Yes	No
37M	Yes	Moderate	18	Yes	2	No	No	No
41F	Yes	Moderate	14	Yes	3	No	No	No
47F	No	Moderate	20	Yes	0	No	No	No
48F	Yes	Severe	9	Yes	4.3	No	No	No
44M	Yes	Mild	8	Yes	12	No	No	No
29F	No	Severe	18	Yes	4	No	Yes	No
59F	Yes	Moderate	25	Yes	3	No	No	No
40M	Yes	*NA*	24	No	0	No	No	No
45M	Yes	Moderate	25	Yes	2.6	No	No	No
20M	Yes	*NA*	1	Yes	4.5	No	No	No
35M	Yes	*NA*	18	Yes	8.8	No	No	Yes
34M	Yes	Mild	8	No	0	No	No	No
37M	Yes	Severe	27	Yes	2.3	No	Yes	Yes
63F	No	Moderate	30	Yes	1.5	No	Yes	Yes
62M	No	Severe	30	No	0	No	No	No
54M	No	*NA*	40	Yes	3	No	No	No
48F	No	Moderate	19	Yes	4.2	No	No	No
43M	Yes	Moderate	24	Yes	0.8	No	No	No
42F	No	Moderate	*NA*	Yes	6.4	No	Yes	Yes
40M	No	Severe	30	Yes	3	No	Yes	No
58M	No	*NA*	20	Yes	4.5	No	No	No
30M	Yes	Moderate	*NA*	No	0	No	No	No
62M	Yes	Severe	25	Yes	5.7	No	No	No
mean ± SD 43.9±11.1/ 8F, 16M								

Table 13: Mood disorder-related information for unaffected control subjects

case	Suicide	Depression severity	Duration of MDD (yrs.)	Antidepressants in blood at death	Antidepressants/ month (grams)	Mania	Psychosis	Antipsychotics
**UNAFFECTED CONTROLS**								
62F	No	None	0	No	0	No	No	No
54M	No	None	0	No	0	No	No	No
52M	No	None	0	No	0	No	No	No
30M	No	None	0	No	0	No	No	No
48M	No	None	0	No	0	No	No	No
51F	No	None	0	No	0	No	No	No
49F	No	None	0	No	0	No	No	No
38F	No	None	0	No	0	No	No	No
44F	No	Mild	0	No	0	No	No	No
44M	No	None	0	No	0	No	No	No
28M	No	None	0	No	0	No	No	No
42F	No	Mild	0	No	0	No	No	No
31M	No	None	0	No	0	No	No	No
51M	No	None	0	No	0	No	No	No
29F	No	Mild	0	No	0	No	No	No
35M	No	None	0	No	0	No	No	No
49M	No	None	0	No	0	No	No	No
17M	No	None	0	No	0	No	No	No
51M	No	None	0	No	0	No	No	No
59M	No	None	0	No	0	No	No	No
mean ± SD 43.2±11.42/ 7F, 13M								

Tables 13-16: Other relevant demographic information for all diagnostic groups

Table 13: Other relevant information for subjects with substance use disorders

case	Anxiety disorders	Personality disorders	Obsessive compulsive disorder	Lithium	Lithium/ mo (grams)	Calcium channel blockers	Antipsychotics last month of life (CPZ eq./grams)	Valproic acid last month of life (grams)
				**SUBSTANCE USE DISORDERS**				
62F	None	Antisocial	No	None	0	No	0	0
54M	None	Antisocial	No	None	0	No	0	0
52M	None	Antisocial	No	None	0	No	0	0
30M	None	None	No	None	0	No	0	0
48M	None	None	No	None	0	No	0	0
51F	None	Antisocial	No	None	0	No	0	0
49F	None	Antisocial	No	None	0	No	0	0
38F	None	Antisocial	No	None	0	No	0	0
44F	None	Antisocial	No	None	0	No	0	0
44M	None	None	No	None	0	No	0	0
28M	None	None	No	None	0	No	0	0
42F	PTSD	Antisocial	No	None	0	No	0	0
31M	GAD	Borderline/ Antisocial	No	None	0	No	6	0
51M		Antisocial	No	None	0	No	0	0
29F	None	None	No	None	0	No	0	0
35M	None	None	No	None	0	No	0	0
49M	None	None	No	None	0	No	0	0
17M	None	None	No	None	0	No	0	0
51M	None	Antisocial	No	None	0	No	0	0
59M	None	Borderline	No	None	0	No	0	0
mean ± SD 34.8±9.0/ 3F, 17M								

Table 14: Other relevant information for subjects with major depressive disorder

case	Anxiety disorders	Personality disorders	Obsessive compulsive disorder	Lithium	Lithium/ mo (grams)	Calcium channel blockers	Antipsychotics last month of life (CPZ eq./grams)	Valproic acid last month of life (grams)
**MAJOR DEPRESSIVE DISORDER**								
43M	None	None	Yes	None	0	No	0	0
63F	None	Schizoid	No	None	0	No	0	0
42M	None	None	No	None	0	No	0	0
50F	None	None	No	None	0	No	0	0
34F	Panic	None	No	None	0	No	1.1	7.5
55M	None	None	No	None	0	No	0	0
33M	None	Dependent	No	Yes	12	No	6.8	0
53M	None	None	No	None	0	*NA*	0	0
44F	None	None	No	None	0	No	0	0
59M	GAD	None	No	None	0	No	0	0
46M	None	Schizoid	No	None	0	No	0	0
36F	Panic	None	No	None	0	Yes	0	0
35M	None	None	Yes	None	0	No	0	0
41M	None	None	No	None	0	No	0	0
56F	GAD	None	No	None	0	No	0	0
62M	None	None	No	None	0	Yes	0	0
20M	Adjustment	None	No	None	0	No	0	0
59M	None	None	No	None	0	No	0	0
48M	*NA*	*NA*	*NA*	None	0	No	0	0
61M	None	None	No	None	0	No	0	0
mean ± SD 47±11.4/ 6F, 14M								

Table 15: Other relevant information for subjects with comorbid substance use disorder and major depressive disorder

case	Anxiety disorders	Personality disorders	Obsessive compulsive disorder	Lithium	Lithium/ mo (grams)	Calcium channel blockers	Antipsychotics last month of life (CPZ eq.)	Valproic acid last month of life (grams)
				**COMORBID SUD AND MDD**				
36M	None	None	No	None	0	No	0	0
37M	None	Dependent	No	None	0	No	0	0
41F	None	None	No	None	0	No	0	0
47F	Phobia	Histrionic	No	None	0	No	0	0
48F	None	None	No	None	0	No	0	0
44M	PTSD	None	No	None	0	No	0	0
29F	None	Borderline	No	None	0	No	0	0
59F	Panic	None	No	None	0	No	0	0
40M	None	None	No	None	0	No	0	0
45M	None	Borderline	No	None	0	No	0	0
20M	GAD	None	No	None	0	No	0	0
35M	None	Mixed	No	None	0	No	2	0
34M	None	Borderline	No	None	0	No	0	0
37M	None	Borderline	No	None	0	No	1.8	0
63F	None	Dependent	No	None	0	No	0.9	0
62M	None	Borderline	No	None	0	No	0	0
54M	None	None	No	None	0	No	0	0
48F	None	None	No	None	0	No	0	0
43M	None	None	No	None	0	No	0	0
42F	None	None	No	None	0	No	3.6	0
40M	None	Dependent	No	None	0	No	0	0
58M	None	None	No	None	0	No	0	0
30M	None	Borderline	No	None	0	No	0	0
62M	*NA*	*NA*	*NA*	None	0	No	0	0
mean ± SD 43.9±11.1/ 8F, 16M								

Table 16: Other relevant information for unaffected control subjects

case	Anxiety disorders	Personality disorders	Obsessive compulsive disorder	Lithium	Lithium/ mo (grams)	Calcium channel blockers	Antipsychotics last month of life (CPZ eq.)	Valproic acid last month of life (grams)
**UNAFFECTED CONTROLS**								
62F	*NA*	*NA*	*NA*	None	0	No	0	0
54M	None	None	Yes	None	0	No	0	0
52M	*NA*	*NA*	*NA*	None	0	No	0	0
30M	None	None	No	None	0	No	0	0
48M	None	Schizoid	No	None	0	No	0	0
51F	None	None	Yes	None	0	No	0	0
49F	*NA*	*NA*	*NA*	None	0	No	0	0
38F	*NA*	*NA*	*NA*	None	0	No	0	0
44F	None	None	Yes	None	0	*NA*	0	0
44M	None	None	Yes	None	0	No	0	0
28M	None	None	No	None	0	No	0	0
42F	None	None	Yes	None	0	No	0	0
31M	*NA*	*NA*	*NA*	None	0	No	0	0
51M	*NA*	*NA*	*NA*	None	0	No	0	0
29F	None	None	No	None	0	No	0	0
35M	None	None	No	None	0	Yes	0	0
49M	None	Antisocial	No	None	0	No	0	0
17M	*NA*	*NA*	*NA*	None	0	No	0	0
51M	None	Paranoid	No	None	0	No	0	0
59M	None	None	Yes	None	0	No	0	0
mean ± SD 43.2±11.42/ 7F, 13M								

***NA* = information not available**
GAD = generalized anxiety disorder
PTSD = post-traumatic stress disorder
