# Supplementary figures and images for: Altered expression of somatostatin signaling molecules and clock genes in the hippocampus of subjects with substance use disorder
Source: Front Neurosci. 2022 Sep 7;16:903941. doi: 10.3389/fnins.2022.903941 (PMC9489843; doi:10.3389/fnins.2022.903941)

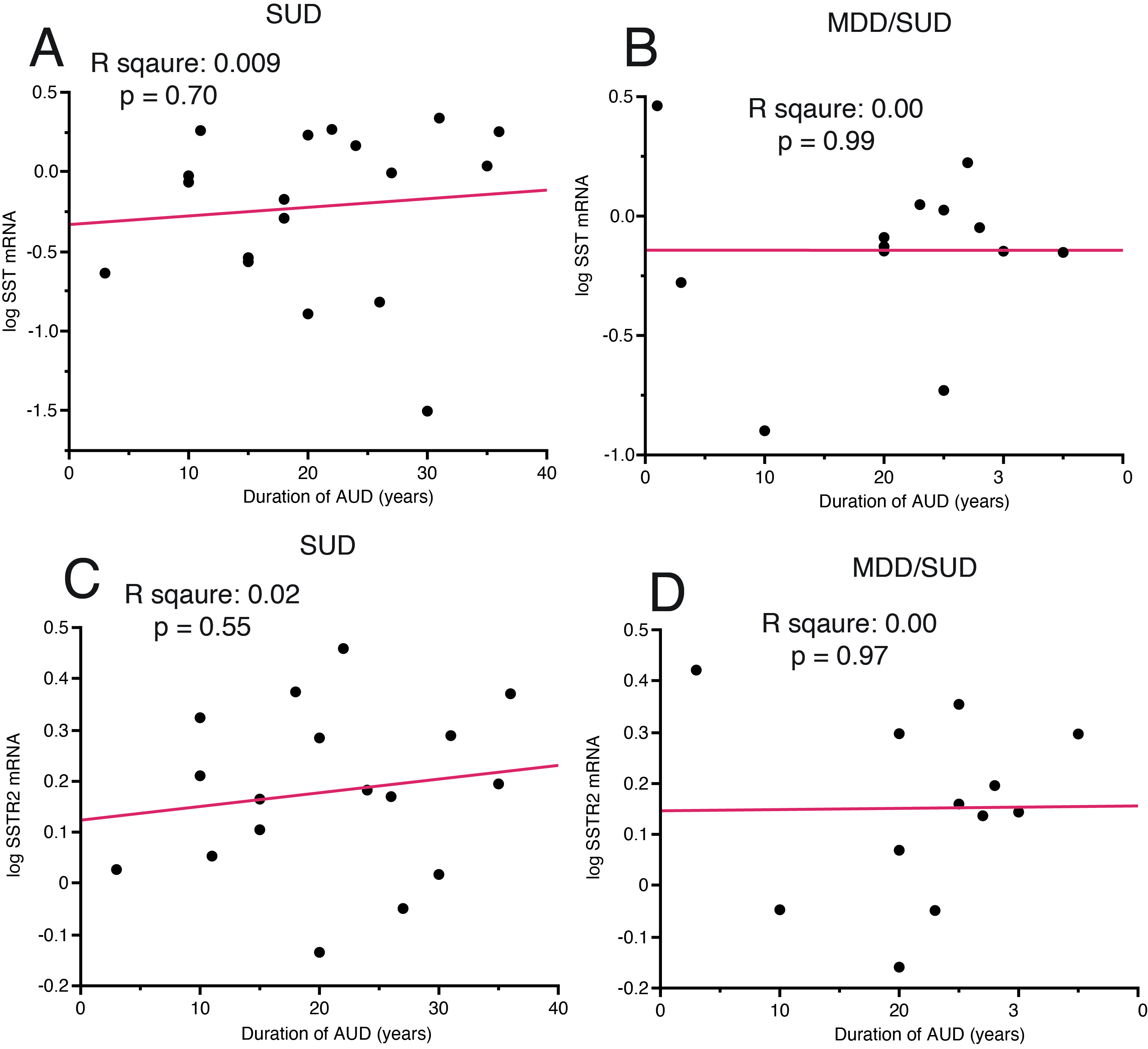

Supplement: Supplementary Figure 1 — Duration of alcohol use disorder is not correlated with expression of SST or SSTR2. Duration of alcohol use disorder was not correlated with SST expression in subjects with SUD (A) or MDD/SUD (B). Similarly, no correlation was observed for duration of alcohol use disorder with expression of SSTR2 in subjects with SUD (C) or subjects with MDD/SUD (D). [file Image_1.JPEG]
